# Graded bulk-heterojunction enables 17% binary organic solar cells via nonhalogenated open air coating

**DOI:** 10.1038/s41467-021-25148-8

**Published:** 2021-08-10

**Authors:** Ying Zhang, Kuan Liu, Jiaming Huang, Xinxin Xia, Jiupeng Cao, Guangming Zhao, Patrick W. K. Fong, Ye Zhu, Feng Yan, Yang Yang, Xinhui Lu, Gang Li

**Affiliations:** 1 Department of Electronic and Information Engineering, Research Institute for Smart Energy (RISE), The Hong Kong Polytechnic University, Hong Kong, China; 2 Department of Physics, The Chinese University of Hong Kong, Hong Kong, China; 3 Department of Applied Physics, The Hong Kong Polytechnic University, Hong Kong, China; 4 Department of Materials Science and Engineering, UCLA, Los Angeles, CA USA

**Keywords:** Renewable energy, Materials for devices

## Abstract

Graded bulk-heterojunction (G-BHJ) with well-defined vertical phase separation has potential to surpass classical BHJ in organic solar cells (OSCs). In this work, an effective G-BHJ strategy via nonhalogenated solvent sequential deposition is demonstrated using nonfullerene acceptor (NFA) OSCs. Spin-coated G-BHJ OSCs deliver an outstanding 17.48% power conversion efficiency (PCE). Depth-profiling X-ray photoelectron spectroscopy (DP-XPS) and angle-dependent grazing incidence X-ray diffraction (GI-XRD) techniques enable the visualization of polymer/NFA composition and crystallinity gradient distributions, which benefit charge transport, and enable outstanding thick OSC PCEs (16.25% for 300 nm, 14.37% for 500 nm), which are among the highest reported. Moreover, the nonhalogenated solvent enabled G-BHJ OSC via open-air blade coating and achieved a record 16.77% PCE. The blade-coated G-BHJ has drastically different D-A crystallization kinetics, which suppresses the excessive aggregation induced unfavorable phase separation in BHJ. All these make G-BHJ a feasible and promising strategy towards highly efficient, eco- and manufacture friendly OSCs.

## Abstract (web summary)

Graded bulk-heterojunction organic solar cell with well-defined vertical phase separation has the potential to surpass the classical counterpart, thus the optimisation of this structure is crucial. Here, the authors reveal solvent selection strategies for optimising morphology of the structure, enabling efficient, eco-friendly, and scalable solar cells.

## Introduction

Bulk heterojunction (BHJ) organic solar cells (OSCs) have attracted extensive attention due to their unique advantages in making flexible and roll-to-roll solar cells via solution processed coating techniques^1–5^. The great progress has been made in the power conversion efficiencies (PCEs) thanks to the rapid development of nonfullerene acceptors (NFAs)^6–11^. In particular, the successful breakthroughs in Y6 series NFAs boosts the single junction BHJ OSCs efficiencies over 17–18%^12–16^. The BHJ strategy, however, has obvious intrinsic limitations for further improvement and technology deployment: (i) in laboratory spin coating optimization, the morphology evolution of BHJ photoactive layer during donor (D) and acceptor (A) mixture is complicated kinetically, which requires delicate balance of treatment conditions, such as D/A ratio, processing solvent and additive, thermal and/or solvent annealing^17,18^. The balance between the phase separation and phase purity remains extremely challenging for the sake of optimal charge separation and charge transport. The vertical components distributions remain uncertain in the present trial-and-error optimization, let alone to control with precision^19^. (ii) The BHJ performances depend strongly on the phase separation, which is closely related to the miscibility and solubility of donor and acceptor in the blend solution and thus limits the selection of processing solvents^20,21^. Major high-performance BHJ OSCs are processed using toxic solvents, such as chlorobenzene (CB) and chloroform (CF)^22,23^. The efficiencies of BHJ OSCs using nonhalogenated low-toxic solvents still lag behind the toxic solvent processed ones^24^; (iii) Due to the short diffusion length and amorphous properties of photovoltaic materials, charge transport pathways can be obstructed once thickening the BHJ film, which is originated from the increased trap densities in the D/A mixed regions with unfavorable phase separation close to the electrodes. Thus, fill factors (FFs) cannot be well maintained in the thick BHJ active layers, leading to the degradation of performances in thicker OSCs^25,26^. (iv) Moreover, when transferring the coating process to the blade coating, the BHJ morphology regulation becomes more complex^27,28^. Spin coating is a fast film drying process enabled by rapid airflow due to the centrifugal effect. During the blade coating process, the slow drying of donor and acceptor mixture wet film makes very different crystallization and intermixing dynamics, leading to totally different D-A phase separation morphologies and thus dramatic variation in the device performances^29–31^.

Solar cell technology started as sharp p–n junction structure in highly crystalline semiconductors. This structure does not work in low mobility and poor crystalline solar cell systems like amorphous silicon (a-Si), where p-i-n structure dominates. Polymeric OSC with revolutionary BHJ structure represents the other extreme of solar cell structures. The concept of gradient junction has also been proposed in solar cell field. After introducing wider bandgap a-SiC:H emitter for p-i-n heterojunction a-Si solar cell, grading p-i interface further advanced a-Si solar cell to 12% PCE. In GaAs solar cell, via varying Al fraction in AlxGa1-xAs, compositional grade in the p layer can be achieved with GaAs near the pn junction, to a wider bandgap alloy at the front window layer surface. The gradient strategy can introduce electric field assisting electron migration, improve minority carrier collection and thus enhance the photocurrent^32^.

The historical technology progresses motivate us to take serious effort integrating controllable gradient concept with BHJ—forming graded BHJ (G-BHJ) realized by sequential deposition (SD) method. In this method, we deposit the D and A layers sequentially, enabling optimization and regulation of the D and A individually with well-maintained crystallinity of materials^33,34^. Furthermore, the D layer and A layer are processed separately, leaving large freedom for the selection of processing solvents, especially considering the use of green solvents, e.g., toluene, *o*-xylene (XY) and tetrahydrofuran (THF), etc. towards high-performance OSCs^34–36^. SD is well utilized in the fullerene based OSCs to construct the comparable efficiencies to the BHJ OSCs, wherein dichloromethane (DCM)/1,2-Dichlorobenzene (*o*-DCB) solvents or DCM/CF are chosen as ideal orthogonal solvents to process fullerene derivatives and polymer donor layer, respectively, due to the distinct solubility of fullerenes and polymers in processing solvents. Consequently, with higher A concentration near the cathode and more D composition at the anode, the vertical graded D-A distributions are more amenable to forming so-called p-i-n structure^37–39^. On the other hand, since small molecular NFAs have similar backbones to polymer donors, the solubility in common solvents is similar, leading to the limited selection of orthogonal solvent as well as the insufficient D/A mixed interfaces formation^40,41^. Although high-performance NFAs-based OSCs via SD method have been previously reported^42–45^, it lacks a clear and quantitative morphological picture. The absence of a rational guideline for solvent selection so far makes the fundamental mechanism underlying the G-BHJ film formation ambiguous. This work aims at removing the scientific ambiguity. When transferring the processing from laboratory spin coating to blade coating film-forming dynamics will be slow, and thus leads to the dramatically different active layer morphology with excessive aggregation of the printed active layer, especially for the NFAs, leading to the oversized domains in the blade coated active layer^46^. To solve this issue, great efforts, such as vacuum annealing, processing solvents engineering, novel material design etc., are made to facilitate the solidification towards reasonable scale of phase separation in high performance OSCs^27,31,47,48^. However, these optimization strategies are complicated and trial-and-error processes, and there are very few works reported in non-halogenated processed highly efficient blade-coated OSCs.

In this work, a simple yet effective G-BHJ formation strategy is demonstrated based on benchmark polymer PM6 donor and BTP-eC9 NFA OSC system. Optimal G-BHJ films from two solvents—XY and CF—are achieved and quantitatively studied by the depth-profiling X-ray photoemission spectroscopy (DP-XPS) technique. An indicative solvent guideline for achieving G-BHJ is provided based on tailored quasi-solid-state inter-diffusion of the two materials. Consequently, the optimal spin-coated G-BHJ OSCs processed by CF and XY deliver outstanding PCEs of 17.54% and 17.48%, respectively. In particular, the XY-based G-BHJ OSC affords a remarkable short-circuit current density (*J*_SC_) of 26.65 mA cm^−2^ and an FF of 0.781, which is one of highest binary OSCs from nonhalogenated solvent. Combined with angle-dependent grazing incidence X-ray diffraction (GI-XRD) measurements, gradient polymer composition and crystallinity distributions are quantitatively visualized: less polymer and weaker crystallinity at the cathode side, while more polymer and stronger crystallinity at the anode side, (and vice versa for acceptor). Noticeably, we found that the G-BHJ can maintain decent PCEs over 14% when the thickness of the active layers is varied in a wide range from120 to 500 nm, which is rarely studied in the G-BHJ studies to the best of our knowledge. Thickness insensitive fabrication is much preferred in roll-to-roll printing. More importantly, nonhalogenated solvent enabled G-BHJ OSC via blade coating to achieve a record 16.77% PCE under open air condition, accompanied by an open-circuit voltage (*V*_OC_) of 0.836 V, a *J*_SC_ of 26.26 mA cm^−2^ and an FF of 0.764. Furthermore, the excessive aggregation induced unfavorable phase separation due to the slow film drying in the conventional BHJ active layer was relieved in the blade-coated G-BHJ film, making G-BHJ strategy a feasible and promising strategy towards highly efficient, eco, and manufacture friendly OSCs.

## Results

### Solvent selection rules and G-BHJ formation

**Fig. 1:**
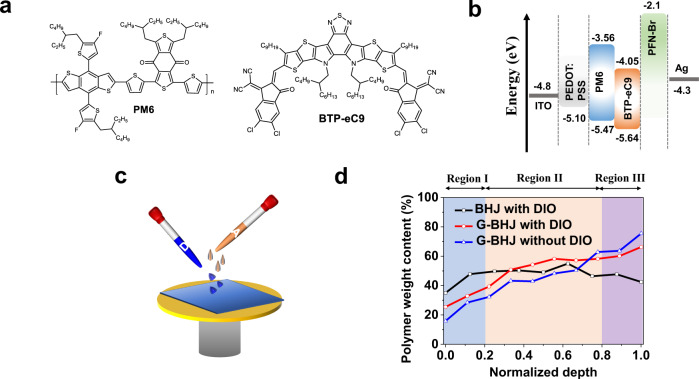
Materials, optoelectronic properties and vertical composition distribution in BHJ and G-BHJ films. **a** Chemical structure of PM6 and BTP-eC9. **b** Schematic illustration of the conventional device structure of OSCs. **c** Schematic diagram of the sequential deposition spin coating procedure. **d** Variation of polymer weight content of BHJ with DIO, G-BHJ without DIO and G-BHJ with DIO films throughout the whole film.

The chemical structures of the polymer donor PM6 and the small molecular acceptor (SMA) BTP-eC9 are shown in Fig. 1a. A conventional structure of indium tin oxide (ITO)/poly(3,4-ethylenedioxythiophene):polystyrene sulfonate (PEDOT:PSS)/active layer/PFN-Br/Ag was adopted here and displayed in Fig. 1b. For sequential processing (Fig. 1c), the solution of neat donor PM6 was firstly deposited on the PEDOT:PSS layer, followed by depositing the neat acceptor solution on top. The solvent properties of upper layer are essential to morphology of the whole blend^49^. Solvent properties including volatile/evaporation rate (boiling point) and solubility (acceptor and donor) need to be taken into consideration to construct successful SD films. Firstly, we varied the processing solvents of upper layer to fabricate SD films: THF, XY, CF, and CB with boiling points of 66 °C, 140 °C, 62 °C and 132 °C, respectively. As shown in the optical images in Supplementary Fig. 1, SD-films processed by CF and XY were visually uniform and smooth, while the SD-film from THF solution was grainy and rough. Pinholes are clearly seen in the SD-film processed by CB due to the destruction of PM6 layer. In terms of solubility, CB and CF can easily dissolve SMA at room temperature, while SMA is found to be poorly soluble in THF and XY at room temperature, and thus high temperatures (60 °C and 80 °C) are needed to dissolve it. Overall, solvent with poor solubility but fast evaporation like THF makes it hardly to achieve a high-quality SD-film. Similarly, solvent with excellent solubility and slow evaporation rate like CB cannot enable high-quality SD-film neither. To further confirm the diffusion of acceptor into donor layer, the donor layer PM6 was washed by same amount of XY solvent, and the GI-XRD technique was used to study the film. Although weaker, the scattering signals maintained characteristic scattering peaks (Supplementary Fig. 2) well, indicative of the XY solvent wash does not disturb the microstructure of PM6 much.

Aiming at elucidating the mechanism, we conducted DP-XPS measurements to quantitatively investigate vertical composition distributions by detecting the atomic ratios throughout the whole SD films^37^. All the samples were prepared on ITO/PEDOT:PSS substrates, as the same as the real devices. Since both PM6 and BTP-eC9 contain oxygen (O), while only PM6 contains fluorine (F), we attribute the O element to both PM6 and BTP-eC9, F atoms to only PM6. One repeat unit of PM6 contains two F atoms and two O atoms, and one BTP-eC9 molecule contains two O atoms. Based on the O/F atom ratio, the polymer weight content (wt%) at different depths can be calculated (see more details in Supplementary Table 1 and Supplementary Note 1)^50^. As shown in Fig. 1d, the conventional BHJ film with DIO displayed a small portion of donor polymer (~45 wt%) at the bottom region, which are generally considered to be negative for charge extraction at the anode. Overall, both SD films with and without DIO treatment display a clear graded polymer distribution from the top surface to the bottom: BTP-eC9-enriched at the top, and PM6-enriched at the bottom, which is clearly distinguished from the BHJ film’s vertical polymer distribution. This proves that the SD method creates a graded BHJ-like morphology, conceptually proposed as “G-BHJ” here.

In the G-BHJ films deposited with and without DIO additive, in addition to the BTP-eC9-enriched at the top (region I), PM6-enriched at the bottom (region III), interestingly there is a bulk region with less variation (region II) in between. For the G-BHJ without DIO, a pronounced gradient composition distribution of polymer from the top region I (15.8 wt%), to the bottom region III (75.5 wt%) was observed. The concentrated PM6 at region III suggests that the weaker interdiffusion can proceed to the deeper region of PM6 layer. While after adding DIO, more polymer contents are located at the bulk region II accompanied by less polymer content (66.4 wt%) enriched at region III, implying that the DIO additive could assist the downshift movement of BTP-eC9 acceptor molecules into PM6 domains. This represents a new morphology manipulation approach compared to those in traditional BHJ. Using the same DP-XPS technique, for SD-films processed by chlorinated solvent (CF), the results verified its graded vertical composition distribution derived from the increasing polymer weight vertically from region I to region III (see Supplementary Fig. 3 and Supplementary Table 2), which matches with the previous CF-based SD studies^51^. To further confirm G-BHJ morphology, time-of-flight secondary-ion mass spectrometry (ToF-SIMS) profiling technique was applied to monitor the vertical profile of donor and acceptor by taking XY-based G-BHJ film (with DIO) as an example (on PEDOT:PSS too, Supplementary Fig. 4). The signal of CN^−^ showed that the BTP-eC9 contents were obviously enriched at the top region, and then decreased gradually to the middle bulk region. The PM6 concentration shows an obvious plateau in the middle part followed by a continuously increasing trend at the bottom region. These results are well consistent to the DP-XPS results wherein G-BHJ morphology is solidly shaped.

The results above provide solid evidence of two successful yet distinct pathways towards valid G-BHJ morphology: (1) high boiling point solvent features longer time for interdiffusion between A and D, yet limited solubility indicates smaller penetration rate of A, leading to proper degree of A’s interdiffusion into D domain (XY-case); (2) low boiling point solvent enables shorter time for A to penetrate downward, yet good solubility provides larger penetration rate of A, reaching similarly functional interdiffusion of A into D domain (CF-case). Hence, the vertical composition profiles of A and D can be finely regulated by the penetration rate and time. Overall, by delicately tailoring the balance of solubility and boiling point of upper solvent, G-BHJ would be expected to be formed. Additive engineering provides a further dimension of morphology manipulation as mentioned before. This offers a simple guideline of solvent selection rule for rational G-BHJ morphology formation by tuning the interdiffusion between A and D.

### Device performance

Based on this G-BHJ concept, we fabricate the OSCs to evaluate the photovoltaic performances for both traditional BHJ and G-BHJ OSCs. For the XY-processed G-BHJ OSCs, the absorption profile was displayed in Supplementary Fig. 5. Optimizing the thickness of donor and the acceptor layer was done by changing spin speed and the 1,8-diiodooctane (DIO) additive amount (See Supplementary Fig. 6 and Supplementary Tables 3–5). With the optimized D/A thickness of 70/55 nm and 0.5% DIO, the best-performing G-BHJ device delivered maximum PCE of 17.48% with a short-circuit current density (*J*_SC_) of 26.65 mA cm^−2^, an open-circuit voltage (*V*_OC_) of 0.840 V, and an FF of 0.781. The fabrication of BHJ was provided in Supplementary Methods. The BHJ reference device, as shown in Table 1, afforded an optimized PCE of 16.41% with a *J*_SC_ of 25.75 mA cm^−2^ and an FF of 0.760. As expected, the optimal G-BHJ device from CF solution exhibited a higher PCE of 17.54% in comparison to BHJ with a PCE of 17.08% (see Supplementary Fig. 7 and Supplementary Table 6). We also study the performances of THF-based and CB-based SD devices, as well as their BHJ counterparts as a comparison (Supplementary Fig. 8 and Supplementary Table 7). The inferior performances of THF-based and CB-based SD devices compared to their BHJ counterparts are due to the failure of constructing effective G-BHJs. For comparison, the BHJ devices made from CB (17.58%), CF (17.08%), and XY (16.41%) all give relatively good performance, while THF completely fails in fabricating BHJ device due to solubility issue, especially in donor polymer. The fact that SD device processed by THF still enables 9.68% PCE further indicates the bigger freedom in constructing OSCs. With the distinct advantage of nonhalogenated solvent processed G-BHJ OSC in the future practical application, we focus on XY processed G-BHJ OSCs from now on.

**Table 1:** Device performances of BHJ and G-BHJ OSCs via spin coating and blade coating processes under the illumination of an AM 1.5 G solar simulator, 100 mW cm^−2^.

	*V*_OC_ (V)	*J*_SC_ (mA cm^−2^)	FF	PCE_max_ (PCE_avg_)^a^ (%)	*J*_calc._^b^
BHJ^c^	0.839 (0.838 ± 0.002)	25.75 (25.59 ± 0.24)	0.760 (0.755 ± 0.010)	16.41 (16.20 ± 0.11)	25.30
BHJ^d^	0.835 (0.835 ± 0.003)	25.24 (25.27 ± 0.15)	0.753 (0.747 ± 0.005)	15.87 (15.78 ± 0.06)	24.92
G-BHJ^c^	0.840 (0.838 ± 0.002)	26.65 (26.55 ± 0.42)	0.781 (0.772 ± 0.008)	17.48 (17.19 ± 0.20)	26.04
G-BHJ^d^	0.836 (0.835 ± 0.004)	26.26 (26.05 ± 0.33)	0.764 (0.756 ± 0.007)	16.77 (16.45 ± 0.16)	25.30

The errors are defined as standard deviation.

^a^Average PCE from ten independent cells.

^b^denotes integrated *J*_sc_ from the EQE curves.

^c^The optimal OSCs via spin coating.

^d^The optimal OSCs via blade coating.

**Fig. 2:**
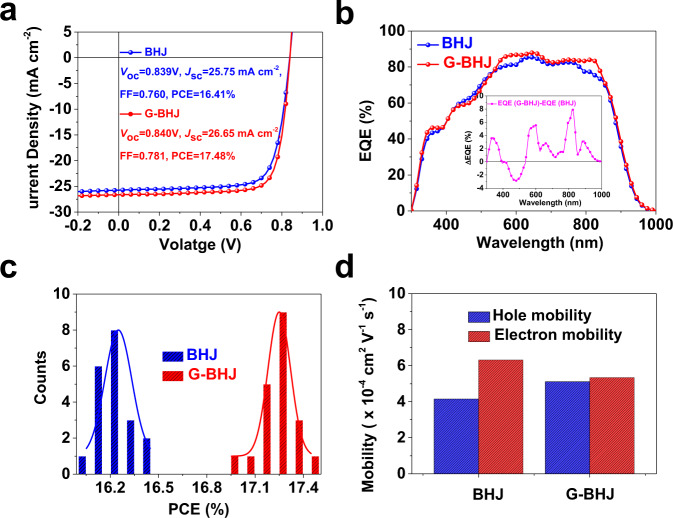
Device performances. **a***J*–*V* curves and **b** EQE response of best-performing BHJ and G-BHJ-based devices. **c** Histograms of the PCE counts for 20 optimal BHJ- and 20 optimal G-BHJ-based devices. **d** Histogram of hole and electron mobilities for BHJ and G-BHJ blends determined by SCLC measurements.

The current density–voltage (*J*–*V*) characteristics of the optimal devices and corresponding performance parameters are depicted in Fig. 2a and Table 1. The PCEs of G-BHJ OSCs outperform the corresponding BHJ control device, with synergistically enhanced *V*_OC_, *J*_SC_, and FF. It is worth-noting that the trade-off between the *J*_SC_-FF was found to be effectively alleviated in the G-BHJ film. Furthermore, the efficiencies processed by nonhalogenated solvent is approaching to that processed from CF solvents in the previous reports^13^. The external quantum efficiency (EQE) of the optimal BHJ and G-BHJ based devices were displayed in Fig. 2b. The integrated *J*_SC_ values of BHJ and G-BHJ OSCs calculated from the EQE curves are 25.30 mA cm^−2^ and 26.04 mA cm^−2^, respectively, as listed in Table 1, which are consistent to those obtained from the *J*–*V* curves (within 2.5% error). Compared with the BHJ devices, the EQE values of G-BHJ devices were enhanced, mainly due to the improvement in the wavelength region corresponding to the PM6 and BTP-eC9 absorption with the highest EQE of 88% at ~650 nm, which can be clearly seen from the EQE differences between BHJ devices and G-BHJ devices. Figure 2c demonstrates the efficiency histograms of BHJ and G-BHJ OSCs.

### Carrier dynamics analysis

The improved *J*_SC_ and FF are the main factors that contribute to the enhanced PCEs of G-BHJ OSCs. To better understand the exciton dissociation and charge collection mechanism in the active layers, the photocurrent density (*J*_ph_) as a function of effective voltage (*V*_eff_) was plotted for the optimal BHJ and G-BHJ OSCs, as plotted in Supplementary Fig. 9. Here, *J*_ph_ = *J*_L_ – *J*_D,_ where *J*_L_ and *J*_D_ are the current densities under illumination and in the dark, respectively. *V*_eff_ = *V*_0_ – *V*_a,_ where *V*_0_ is the voltage when *J*_L_ equals *J*_D_ and *V*_a_ is the applied voltage. On the basis of this curve, the ratio of *J*_ph_/*J*_sat_ (the saturation *J*_ph_) under the short-circuit condition relates to exciton dissociation efficiency (*η*_diss_), and the charge collection efficiency (*η*_coll_) is evaluated by the ratio of *J*_ph_/*J*_sat_ at the maximal power output point. The related parameters above are provided in Supplementary Table 8. The *η*_diss_ value of the optimized G-BHJ devices is 96.03%, which is slightly larger than that of the BHJ devices. The improved *η*_diss_ and *η*_coll_ for the optimized G-BHJ device demonstrates a better exciton dissociation and charge collection, well explain the synchronous enhancement of *J*_SC_ and FF in the G-BHJ OSCs.

In addition, the slope of ${nkT}/q$ in the function of ${V}_{{{{\rm{OC}}}}}\propto nkT/q{{{\rm{ln}}}}P$ (*k* is Boltzmann constant, *q* is elementary charge, and *T* is temperature) is an indicator of the trap-assisted recombination (monomolecular recombination), which could be extracted by plotting *V*_OC_ versus the natural logarithm of *P* (Supplementary Fig. 10a). The comparable fitted slopes between BHJ (1.24*kT*/*q*) and G-BHJ devices (1.21*kT*/*q*) indicate that the G-BHJ strategy shows similar trap-assisted charge recombination.

With regard to the bimolecular recombination loss, the dependence of *J*_SC_ on the *P* can be described by the power law relationship $({J}_{{{{\rm{SC}}}}}\propto {P}^{{{{\rm{s}}}}})$, where *S* is the exponential factor related to bimolecular recombination. As shown in Supplementary Fig. 10b, the calculated *S* value for the optimal G-BHJ device is approaching unity (0.98), larger than that of BHJ device (0.95), reflecting the weak bimolecular recombination loss under short circuit condition. The reduced charge recombination losses (monomolecular and bimolecular recombination) are assumed to benefit from the expected vertical phase separation with acceptor enriched at the cathode and donor enriched at the anode sides.

The photoluminescence (PL) quenching efficiency was employed to further investigate the charge dissociation properties in different films. As displayed in Supplementary Fig. 11, the neat PM6 and BTP-eC9 exhibited distinct PL spectra with maximal emission peaks located at 682 nm and 936 nm, respectively. Both the BHJ and G-BHJ films exhibit efficient PL quenching. The PL intensities of BHJ and G-BHJ films decrease by 97.6% and 99%, respectively, compared to that of the neat PM6 film (Supplementary Fig. 11a). Similarly, the quenching efficiencies of BHJ and G-BHJ films, relative to the neat BTP-eC9, are calculated to be 94.5% and 98.1%, respectively (Supplementary Fig. 11b). These positive results suggest that the quenching efficiencies of G-BHJ film is even stronger than that of BHJ film, implying that the sufficient D/A interfaces are well formed for efficient charge transfer in the G-BHJ OSCs using non-orthogonal solvents, contributing to the higher *J*_SC_ (26.65 mA cm^−2^).

To explore the improvement of FF in G-BHJ based devices, we investigated the charge transport properties in the BHJ and G-BHJ based devices by fabricating the electron-only diodes using the structure of ITO/ZnO/BHJ (G-BHJ) active layer/Ag and hole-only diodes using the architectures of ITO/PEDOT:PSS/ BHJ (G-BHJ) active layer/Au, respectively. By fitting the *J*–*V* curves in the space-charge-limited current region (SCLC) in Supplementary Fig. 12. The detailed hole mobilities (*µ*_h_) and electron mobilities (*µ*_e_) values are calculated and summarized in Supplementary Table 9. The *µ*_e_ of the optimal BHJ and G-BHJ devices are calculated to be 6.32 × 10^−4^ and 5.34 × 10^−4^ cm^2^ V^−1^ s^−1^, while the *µ*_h_ of them are 4.15 × 10^−4^ and 5.11 × 10^−4^ cm^2^ V^−1^ s^−1^, respectively (Fig. 2d). There are two main factors affecting the charge transport: molecular packing and transportation pathways^50^. The reason of reduced *µ*_e_ and the improved *µ*_h_ in the optimal G-BHJ devices may be due to the strengthening of the polymer crystallinity and reduced crystallization of acceptor, which will be discussed in the GIWAXS part. As a result, a more balanced *µ*_e_/*µ*_h_ of 1.05 was achieved for the G-BHJ devices in comparison with that of 1.52 in the BHJ counterparts, indicative of more balanced charge transport properties and less isolated domains in the optimal G-BHJ devices, leading to less charge recombination and thus higher FF (≈0.78) can be achieved.

### Molecular packing and crystallinity analysis

**Fig. 3:**
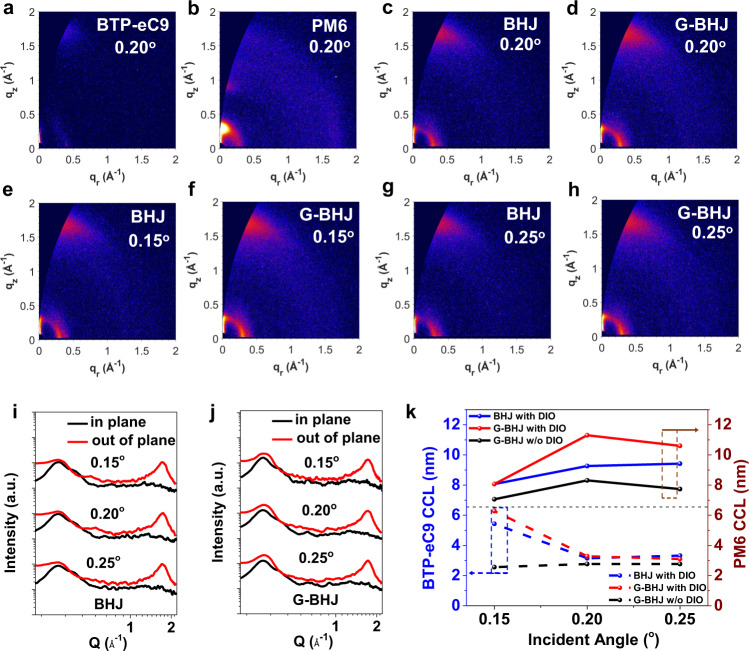
Molecular packing and crystallinity of BHJ and G-BHJ films. 2D GIWAXS patterns of **a** neat BTP-eC9 film, **b** neat PM6 film, **c** the optimal BHJ and **d** the optimal G-BHJ processed films at critical incident angle of 0.20°. 2D GIWAXS patterns of **e** the optimal BHJ and **f** the optimal G-BHJ films collected at incident angles of 0.15°. 2D GIWAXS patterns of **g** the optimal BHJ and **h** the optimal G-BHJ films collected at incident angles of 0.25°. The corresponding 1D linecut profiles of **i** the optimal BHJ and **j** the optimal G-BHJ films at three different X-ray incident angles of 0.15°, 0.20° and 0.25°, respectively. **k** Coherence lengths of PM6 and BTP-eC9 in three films versus varied incident angles.

To further understand the vertical morphology characteristics in the BHJ and G-BHJ active layers, the GI-XRD measurements were performed, giving details of the molecular packing and crystallinity. To give the vertical crystallinity information across the whole film, angle-dependent GI-XRD was performed in this study. The functional relationship between theoretical penetration depth of X-ray beam and incident angle was depicted in Supplementary Fig. 13. If the incident angle of X-ray (*α*) is smaller than critical angel (*α* < *α*_c_ = ~0.2°), the top ~7 nm (upper layer) of the film can be detected. If *α* > *α*_c_, the whole depth of film can be penetrated, from which we can map the overall crystallinity of the film. The two-dimensional (2D) GI-XRD patterns are shown in Fig. 3a–h and the corresponding line profiles in the in-plane (IP) and out-of-plane (OOP) directions are extracted in Fig. 3i, j. Firstly, critical incident angle of about 0.2° was chosen to reveal the average crystallinity of the neat materials as well as the optimal BHJ and G-BHJ active layers throughout the whole film^52^. The neat BTP-eC9 displayed the preferred face-on orientation with a clear π–π stacking peak at *q* ~ 1.76 Å^−1^ in the OOP direction and a lamellar peak at *q* ~ 0.40 Å^−1^ in the IP direction, which is consistent with the previous reports^13^. The pristine PM6 also adopts the face-on orientation with respect to the substrate, showing a lamellar peak at *q* ~ 0.30 Å^−1^ along the IP direction and a π–π stacking peak (010) at *q* ~ 1.69 Å^−1^ along the OOP direction, respectively. For the blend films, it was found that both BHJ film and G-BHJ film maintained the face-on orientation, indicating the G-BHJ approach does not change the molecular orientation property. Noticeably, both BHJ and G-BHJ films showed two obvious characteristic peaks in the IP direction at lower *q* region, which can be assigned to the PM6 with higher intensity at *q* ~ 0.3 Å^−1^ and BTP-eC9 with lower intensity at *q* ~ 0.4 Å^−1^, respectively. Thanks to this, peak fitting analysis was conducted to reveal the different impacts of BHJ and G-BHJ methods on the packing ordering of individual components. This was done by taking line cuts from the GI-XRD patterns in the IP direction and assigning the IP peaks to individual components. Crystalline coherence lengths (CCL) can be used to estimate the crystal size of the domains, which could be determined using Scherrer equation: CCL = 2π*K*/FWHM, where *K* is a shape factor (here is 0.9) and FWHM is the full width at half-maximum of each peak^53^. The detailed lamellar stacking CCL and π–π stacking CCL data are summarized in the Supporting Information. The calculated CCL of PM6 lamellar stacking (100) (*q* ~ 0.29 Å^−1^) was 92.6 Å in the BHJ film, while the CCL for the G-BHJ film increased to 113 Å, meanwhile the d-spacing of (100) packing decreased (Supplementary Table 10), indicating the ordering of packing for PM6 was enhanced in the G-BHJ film. For the small molecular NFA, it was found that both BHJ and G-BHJ films can induce the lamellar diffraction from BTP-eC9 with similar CCLs (Supplementary Table 10). Furthermore, both BHJ and G-BHJ films possess strong π–π stacking peaks of (010) along the OOP direction, which should belong to the combination of PM6 and BTP-eC9 and difficult to distinguish. The calculated (010) CCLs of BHJ and G-BHJ films are estimated to be 20.6 Å and 19.9 Å (Supplementary Table 11), respectively, showing similar π–π stacking structure. Thus, utilizing non-orthogonal solvent driven G-BHJ approach is demonstrated to facilitate the crystallization structure of PM6, which can be explained by the fact that PM6 is solidified first to form a scaffold and could maintain the pristine nanostructure to certain degree in the XY solvent during the acceptor coating. The enhanced crystallinity of polymer is in excellent agreement with the improved hole mobility as discussed above.

The smaller grazing angle of 0.15° was chosen to detect the crystallization properties at the upper layer (~7 nm). The upper layer GI-XRD results revealed the BTP-eC9 IP lamellar stacking peaks (100) existing in both BHJ and G-BHJ films, indicating the existence of BTP-eC9 crystallites on the top layer. Further peak analysis revealed that the optimal G-BHJ film processed larger lamellar stacking CCL (~62.8 Å) for BTP-eC9 than that of BHJ film (~54.3 Å) (Supplementary Table 10), confirming more ordered BTP-eC9 aggregates formation at the surface of G-BHJ films and thus providing more continuous pathways for electron transport. The crystal sizes of PM6 donor at the top layer, on the other hand, are almost the same. We also chose larger incident angle larger than critical angle (0.25°) to detect the average crystalline structure of the entire film. The CCL of PM6 lamellar stacking increased significantly from the incident angle of 0.15° to 0.25°—from 80.7 Å nm to 102 Å in the G-BHJ film and 80.7 Å to 94.2 Å in the BHJ film (Supplementary Table 10), respectively, indicating larger gradient of PM6 crystalline nanostructure in the G-BHJ film from the top surface to the substrate. On the contrary, the crystal size of BTP-eC9 displayed a decreased trend from the top surface to the bottom substrate, giving the evidence that the aggregation of small molecules can be inhibited when the small molecules diffuse towards the bottom layer. This is reasonable because the interdiffusion processes and crystal nucleuses growth of BTP-eC9 are competitive dynamic processes^42^. Also, the enhanced crystallization of PM6 at the bottom potentially could further restrict the self-assembly of BTP-eC9. Generally, the (010) π–π stacking show the similar trend with lamellar stacking (Supplementary Table 11). The more ordered BTP-eC9 structure on the top and improved crystallinity of PM6 on the bottom in the optimal G-BHJ active layer contributes to the gradient vertical crystallinity towards more valid charge transport properties, leading to the higher *J*_SC_ and FF. Furthermore, we also measured the angle-dependent GI-XRD of the G-BHJ film without additive to elucidate the functional role of DIO on the vertical crystallinity in the G-BHJ film (Supplementary Fig. 14 and Supplementary Tables 10, 11), which is rarely studied in the sequential deposited OSCs. The simultaneously prolonged CCLs for the lamellar stacking of PM6 and BTP-C9 throughout the whole film after the incorporation of DIO, especially the BTP-C9 on the top and PM6 on the bottom, demonstrate that 0.5% DIO could induce the strong growth of crystallites for both polymer and small molecules. Furthermore, the more balanced crystallization between the polymer donor and small molecular acceptor from top to bottom can be clearly observed, which indicates that DIO-assisted G-BHJ strategy could finely reduce the crystallization gap of D-A domain. To assist the visualization, the vertical evolution of crystal size (CCL) for respective PM6 and BTP-eC9 is presented in Fig. 3k. The synergetic effect of G-BHJ (by SD process) and delicately tuned/enhanced D-A crystallinity (by additive) is behind the success of optimal G-BHJ devices.

**Fig. 4:**
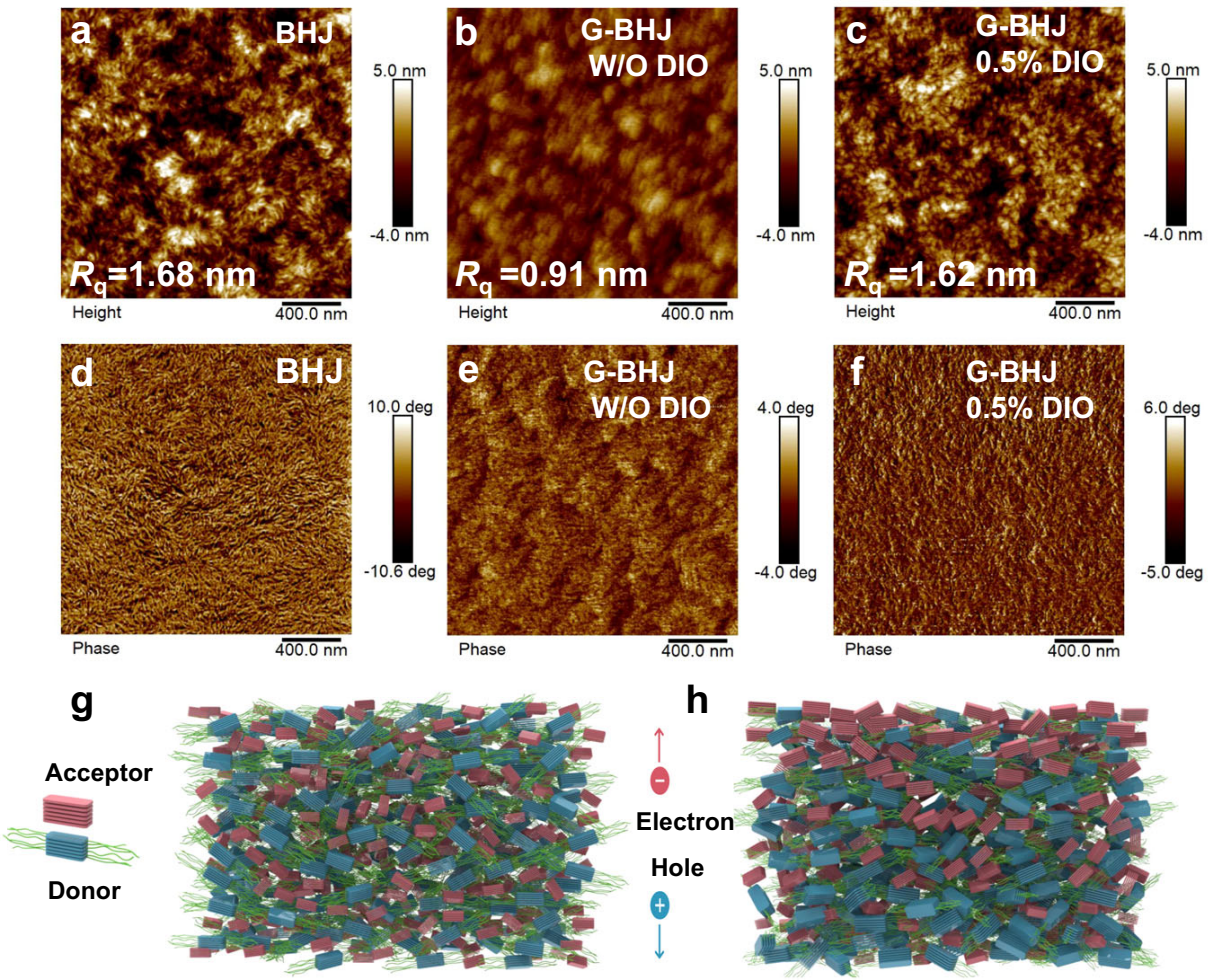
The morphology of BHJ and G-BHJ films. **a**–**c** AFM height images, **d**–**f** AFM phase images of the optimal BHJ film, G-BHJ film without DIO and the optimal G-BHJ film with 0.5% DIO. The schematic morphology of **g** the optimal BHJ film and **h** the optimal G-BHJ film along the vertical direction.

Atomic force microscope (AFM) and transmission electron microscopy (TEM) characterizations were further employed to study the surface and bulk morphologies of blend films. As shown in Fig. 4a–c, AFM images of three films exhibit apparently different phase separation features on the top layer. For example, in the phase images (Fig. 4d–f), the fibril-like aggregation can be clearly observed in the BHJ blend film, which is closely dependent on the crystalline feature of polymer donor PM6, while the surface morphology of the G-BHJ film without DIO tends to form uniformly distributed granular aggregates, which is assumed to be due to the strong crystallization of BTP-eC9. Particularly, after DIO addition, the G-BHJ film shows the possibly more optimal phase separation with appropriate domain size, that features both polymer threadiness and small molecular granule, implying that the DIO additive could assist the upshift movement of PM6 for realizing sufficient intermixed regions. The mean-square surface roughness (*R*_q_) of optimal BHJ film, G-BHJ film without DIO and optimal G-BHJ film are found to be 1.68 nm, 0.91 nm and 1.62 nm, respectively. The AFM results are well consistent with the GIWAXS measurements. The TEM images of the three films exhibit similarity, indicating similar overall D-A phase-separation morphologies in the bulk part (See Supplementary Fig. 15), as TEM gives information of the whole film.

Based on the AFM (on surface) and TEM (on bulk) observations, we infer that the intermixed D/A microstructure is successfully evolved due to the easy swelling of PM6 material in XY when depositing the acceptor. However, the more pronounced vertical phase separations tuned by the G-BHJ method is expected, as evidenced vide supra, which brings about simultaneous gain of charge separation and transport and thus effectively reduce the trade-off between *J*_SC_ and FF. Finally, combined with DP-XPS and morphology results, we capture the picture of the vertical evolution morphology in BHJ and G-BHJ photoactive layers, respectively, as shown in Fig. 4g, h. In G-BHJ, less polymer with weaker crystallinity at the cathode side, while more polymer with stronger crystallinity at the anode side, (and vice versa for acceptor concentration and crystallinity distribution).

### Thick-film G-BHJ OSCs

**Fig. 5:**
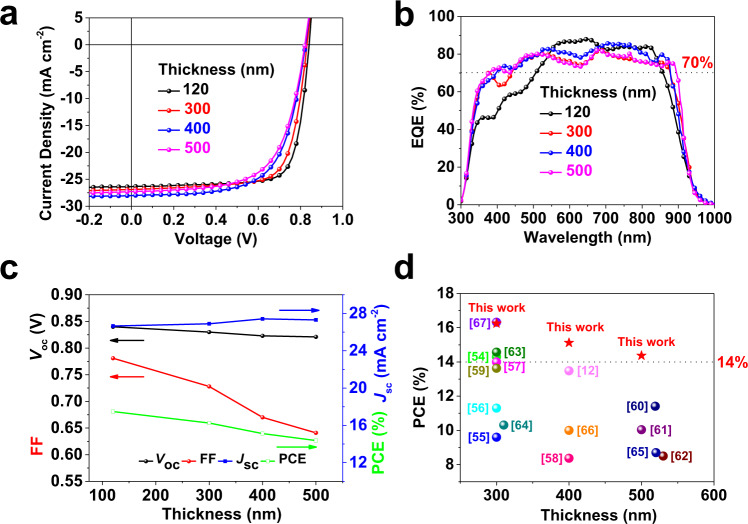
The photovoltaic properties of thick G-BHJ OSCs. **a***J*–*V*, **b** EQE curves of the optimal G-BHJ OSCs with different active layer thickness. **c** device performance parameters (PCE, *J*_SC_, *V*_OC_, and FF) versus the active layer thickness. **d** Comparison of our results with previously reported PCEs and thickness for both binary and ternary OSCs. The detailed material systems and device parameters of each point are provided in Supplementary Table 12.

Inspired by the advantages of more balanced hole and electron mobilities and superior vertical morphological structure in the G-BHJ approach, we explored thick-film OSCs (up to 500 nm) by varying the concentration of donor and acceptor and their spin coating speeds. Noticeably, the thickness effects of donor and acceptor on the photovoltaic performances are rarely studied in the previous sequential deposition literature. The *J*–*V* curves G-BHJ OSCs are displayed in Fig. 5a and the corresponding photovoltaic parameters are summarized in Table 2. Thick-film based BHJ OSCs were also fabricated as comparison and the detailed results are provided in Supplementary Methods. As shown in Fig. 5b, broader and higher EQE spectra are observed for thick OSCs, the integrated *J*_SC_ from EQE spectra are well consistent with the *J*_sc_ measured in *J*–*V* curves within small error (less than 3.5%). As illustrated in Fig. 5c, *V*_OC_ values slightly decreased with the increase of thickness from 120 nm to 500 nm, while *J*_SC_ values increase from 26.65 to 27.42 mA cm^−2^ when the thickness increased from 120 to 400 nm due to the enhanced photon absorption. Although FF has a reduction to some extent for devices thicker than typical 120 nm, it remained the high values of 0.728 for 300 nm and 0.670 for 400 nm, which are outstanding in the previous reported thick-film based OSCs in both binary and ternary systems (Supplementary Table 12). The decrease of FF is partially related to the low mobility of organic photovoltaic materials, leading to the serious bimolecular recombination in the thick active layer^12^. It was worth-noting that G-BHJ OSCs possess higher tolerance to the wide range of thickness up to 500 nm compared to the BHJ OSCs (Supplementary Fig. 16 and Supplementary Table 13), mainly resulting from the superior FFs. For BHJ devices, the FF drops from 0.76 in 110 nm to 0.58 in 500 nm OSC, while in G-BHJ, the drop is much milder—from 0.78 in 120 nm to 0.64 in 500 nm. The well maintained FFs in thick OSCs is also a clear indication that the system has smaller non-geminate recombination in G-BHJ devices. Finally, high efficiencies of 16.25% for 300 nm and 15.12% for 400 nm active layer are realized for G-BHJ OSCs, respectively, which are among the highest values for OSCs with thick active layer (Fig. 5d), including both the binary and ternary OSCs^12,54–67^. To further support the G-BHJ morphology in thick device, DP-XPS characterization was conducted for 500 nm-thick G-BHJ film (Supplementary Fig. 17 and Supplementary Table 14). Overall, the gradient polymer content is found to be more pronounced in thick G-BHJ OSCs (top: 16 wt%; bottom: 90 wt%). Therefore, G-BHJ strategy is promising and feasible to construct highly efficient thick OSCs, critical to the future industrial roll-to-roll production.

**Table 2:** Photovoltaic parameters of the optimal G-BHJ OSCs with different active layer thickness.

Active layer thickness (nm)	*V*_OC_ (V)	*J*_SC_ (mA cm^−2^)	FF	PCE_max_ (PCE_avg_)^a^ (%)	*J*_calc._^b^ (mA cm^−2^)
120 ± 5	0.840	26.65	0.781	17.48 (17.19 ± 0.20)	26.04
300 ± 10	0.830	26.89	0.728	16.25 (16.00 ± 0.23)	26.09
400 ± 10	0.823	27.42	0.670	15.12 (14.92 ± 0.28)	27.11
500 ± 15	0.821	27.31	0.641	14.37 (14.19 ± 0.25)	26.39

The errors are defined as standard deviation.

^a^Average PCE from ten independent cells.

^b^denotes integrated *J*_SC_ from the EQE curves.

### G-BHJ OSCs fabrication by blade coating

**Fig. 6:**
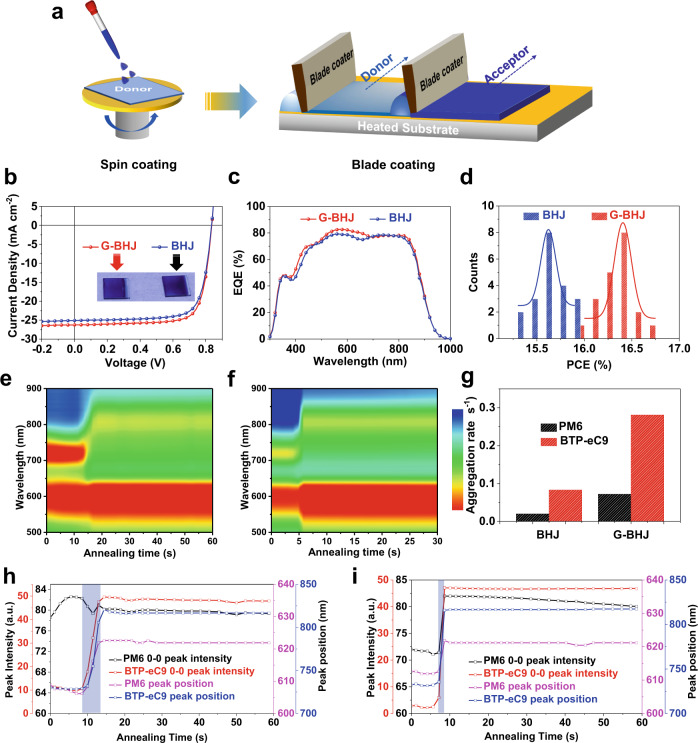
The open-air blade coating device performances and in situ absorption characteristics for both BHJ and G-BHJ OSCs. **a** Schematic illustration of spin coating and blade coating for G-BHJ fabrication procedure. **b** *J*–*V* curves of the BHJ OSCs and G-BHJ OSCs. **c** EQE curves of the optimized BHJ OSCs and G-BHJ OSCs. **d** Histograms of the BHJ OSCs and G-BHJ OSCs based on 20 independent cells. Time-resolved UV-vis absorption spectra of **e** optimized BHJ and **f** G-BHJ OSCs films. **g** Aggregation rate of PM6 and BTP-eC9 in optimized BHJ and G-BHJ active layers. The peak position and peak intensity evolution as a function of annealing time of PM6 and BTP-eC9 in **h** optimized BHJ and **i** G-BHJ films.

Encouraged by the outstanding photovoltaic performances of G-BHJ OSCs via spin coating method, we further employed doctor-blading technique to fabricate both BHJ and G-BHJ OSCs in the same device structure, which is significant for the deployment of large-scale modules (Fig. 6a). In this study, BHJ and G-BHJ active layers was both deposited in open-air (30 ± 5% RH) using air-knife assisted blade-coating technique^68,69^, from non-halogenated XY solvent. The high boiling point of XY (140 °C) will reinforce the aggregation^27,31^, so employing laminar air-knife quenching together with hot-casting substrate at a moderate temperature of 60 °C could well control the drying kinetics of polymer wet film. The active layers of both BHJ and G-BHJ are optimized by controlling DIO contents as well as the coating speeds (see Supplementary Fig. 18 and Supplementary Tables 15, 16). Figure 6b shows the *J*-*V* curves of the optimized blade-coated BHJ and G-BHJ OSCs and the corresponding photovoltaic parameters are listed in the Table 1. The optimal G-BHJ devices exhibited a maximum PCE of 16.77% with a *V*_OC_ of 0.836 V, a *J*_SC_ of 26.26 mA cm^–2^, and an FF of 0.764. For blade-coated BHJ devices, an inferior efficiency of 15.87% was achieved accompanied by a *V*_OC_ of 0.835 V, a *J*_SC_ of 25.24 mA cm^–2^ and an FF of 0.753. The improvement of the photovoltaic performances in G-BHJ based device mainly originates from the enhanced *J*_SC_ and FF, with the same trend as spin-coated devices. The higher *J*_SC_ value in G-BHJ device can be crosschecked with EQE measurements. The EQE spectra of G-BHJ device presented slightly higher photocurrent generation for both donor and acceptor absorption region. As a result, the integrated *J*_SCS_ are calculated from the EQE curves plotted in Fig. 6c, to be 24.92 and 25.30 mA cm^–2^ for BHJ and G-BHJ OSCs, respectively, which matches well with the *J*–*V* measurements. The histograms of PCEs for BHJ and G-BHJ devices are plotted in Fig. 6d. The PCEs of G-BHJ OSCs are distributed within the range of 16.0 to 16.75%, while the related BHJ devices fluctuated in the range of 15.3 to 15.9%. We also investigated the CF-based G-BHJ OSCs via blade coating. As shown in Supplementary Fig. 7 and Supplementary Table 6, the optimal devices can give a high PCE of 16.78%, which indicates the good compatibility of G-BHJ strategy in coating process. It is noteworthy that two other representative polymer/SMA material systems: PM6:IT-4F and PCE-10:IEICO-4F, gave better PCEs of G-BHJ versus their respective BHJ counterparts in both spin coating and blade coating processes, demonstrating the applicability of using G-BHJ strategy in D-A material combinations to promote photovoltaic performance of OSCs (see detailed performances in Supplementary Fig. 19 and Supplementary Table 17).

To understand the surprising enhancement of *J*_SC_ and FF in the G-BHJ devices processed by XY, we then investigated the crystallization kinetics of donor and acceptor. Here, in situ UV–vis absorption measurements were performed to detect the phase transition during the film formation from solution state to film state^70^. The 2D time-resolved UV–vis absorption mapping is displayed in Fig. 6e, f for BHJ and G-BHJ films and the corresponding line profiles are provided in the Supporting Information. Since the absorption peak of donor and acceptor in the blends are clearly distinguishable, we then plotted the peak position and peak intensity of PM6 and BTP-eC9 as a function of annealing time to investigate their individual aggregation evolution (Fig. 6h, i). The 0–0 absorption peaks of small molecule exhibited red-shift tendency from ~731 nm to ~820 nm in both BHJ and G-BHJ films accompanied by a dramatic increase of peak intensity, which implies that the molecular ordering stage of BTP-eC9 occurs (Supplementary Fig. 20). Noticeably, it takes 12.9 s for the BHJ film to form a dry film, while it takes only 8.6 s for the G-BHJ film to be solidified under the same post-treatment condition, implying that the G-BHJ strategy effectively facilitates the film drying process. Interestingly, another distinction between G-BHJ and BHJ films exists in the wet film stage. While the peak intensity of polymer in BHJ film (black curve in Fig. 6h) undergoes an increased trend due to the evaporation of solvent (so called pre-aggregation stage), it is completely suppressed in the G-BHJ film, indicating that the G-BHJ processing strategy affects the aggregation of polymers and thus lead to the distinctive morphological features. We infer that when depositing the second acceptor layer, the bottom polymer layer is actually entering the semi-wet state, thus the pre-aggregation stage is ignored and directly undergoes the film transformation from semi-wet state to the solid state. Furthermore, the aggregation rate of polymer and small molecule was deduced from the slope of the peak and studied, shown in Fig. 6g. It was found that the aggregation rate of small molecule is higher than that of polymer in both BHJ (0.083 s^−1^ for acceptor and 0.020 s^−1^ for donor) and G-BHJ films (0.281 s^−1^ for acceptor and 0.072 s^−1^ for donor). Compared to the aggregation rate of BTP-eC9 in BHJ film, the acceptor in the G-BHJ film can be rapidly aggregated which is indicated by the sharp slope of the peak, almost three times faster than that in BHJ film. We hypothesize that the polymer layer could offer nuclei (or nucleation sites) for the small molecule layer in the G-BHJ process, which could induce the faster ordering of the small molecule layer^29^. It has revealed that the slow solidification leads to longer time for the active layer to aggregate and crystallize, giving oversized domain or excessive phase separation in the active layer^46^. Therefore, the faster film formation process in the G-BHJ provides rapid crystallization time, which prohibits the excessive aggregation of NFA^30^. Moreover, the aggregation rate of polymer in the G-BHJ film also becomes higher in comparison with that in the BHJ film. This faster polymer aggregation rate is associated with the inhibited polymer pre-aggregation stage, which saves time for the semi-solid film to transform directly to the solid film. The more balanced aggregation kinetics between the polymer and small molecule NFA in the G-BHJ blend indicate the more balanced phase domains, which is beneficial to efficient exciton dissociation and charge transport, contributing to higher *J*_SC_ and FF. We also performed AFM measurements to probe the surface morphology of the optimal blade-coated BHJ and G-BHJ films (Supplementary Fig. 21). As expected, small RMS value in the optimal G-BHJ film (1.14 nm) can be observed, confirming the suppressed aggregation behavior in the G-BHJ printed active layer. Therefore, we have validated the non-halogenated solvent enabled G-BHJ method for highly efficient blade-coated OSCs via regulating the crystallization kinetics of both donor and acceptor and thus phase separation in the active layer.

## Discussion

In this manuscript, we demonstrate the G-BHJ morphology modulation in state-of-the-art OSC systems with proposed solvent guidance. Insights of G-BHJs are provided through quantitative investigations of depth-profiling XPS and angle-dependent GI-XRD techniques. The graded composition and crystallinity distribution in principle can benefit the carrier transport, while the graded BHJ still gives sufficient D/A interface. The spin-coated G-BHJ OSCs processed by CF and XY deliver outstanding PCEs of 17.54% and 17.48%, respectively. The G-BHJs’ carrier transport advantage indeed enabled high-performance thick film OSCs from XY-16.25% (FF of 0.728) for 300 nm, 15.12% (FF of 0.670) for 400 nm, and 14.37% (FF of 0.641) for 500 nm thick OSCs. Probably even more excitingly, the nonhalogenated solvent (XY) enabled G-BHJ OSC via blade coating to achieve an excellent 16.77% PCE in open air condition, assisted by the drastically different but favorable novel D–A crystallization kinetics comparing to classical BHJ.

One should remember that these encouraging results were achieved using proper solvent coating twice sequentially (CF and XY as examples here). This is just at the infant stage of a new research direction, and it opens a door of solvent engineering for achieving various G-BHJ with tunable D/A composition and crystallinity gradient profiles. In our opinion, smart selection of “green” solvents with different solubilities to the existing high performance OSC systems and engineering deposition conditions wisely, or even design new OSC D–A molecules fitting “good” solvents are both very valuable for next step G-BHJ OSC research. The scale-up effort is always an important step for the scientific findings to be applicable. The in situ approaches, including the UV–Vis here as well as GI-XRD, electron microscopy etc., are believed to be critical for understanding the solar ink thin film molecular packing, morphology evolution and structure-property relationship. These are challenging tasks, but deserve serious investigations to move the new field forward. Overall, this work shows the G-BHJ reported here is a feasible and promising strategy towards highly efficient, ecofriendly and manufacture friendly OSCs.

## Methods

### Materials

The molecular weight of the PM6 was characterized with high-temperature gel permeation chromatography (GPC) at 160 °C with 1,2,4-trichlorobenzene as solvent, which yields *M*_n_ = 35,800, *M*_w_ = 97,000, PDI = 2.7. BTP-eC9 were purchased from Solarmer Materials Inc. All other chemical reagents were used as received.

### Device fabrication and characterization

Polymer solar cell devices were fabricated in the conventional structure of ITO/PEDOT: PSS/Active layer/PFN-Br/Ag. The substrates were firstly cleaned using detergent, deionized water, acetone, and isopropanol for every 30 min, and then treated in ultraviolet ozone for 20 min. Followed by spin coating a thin layer (~30 nm) of PEDOT:PSS (Bayer baytron 4083) on the precleaned ITO-coated glass substrates at 3000 rpm for 30 s and then annealed at 150 °C for 15 min. Then, the substrates were transferred into a glovebox. Afterwards, for the BHJ devices, the optimized active layer was spin coated from *o*-xylene solution with polymer concentration of 10 mg mL^−1^ (D:A = 1:1.2, 0.5% DIO) at 3500 rpm for 60 s to form an active layer of ~120 nm. Then, the active layer was thermal annealed at 100 °C for 10 min on the hotplate. For the G-BHJ processed device, the PM6 solution was prepared in *o*-xylene at 10 mg mL^−1^ at 60 °C stirring. BTP-eC9 solution was prepared in *o*-xylene at 10 mg mL^−1^ with 0.5% DIO by volume at 80 °C stirring. The PM6 solution was spin-coated on PEDOT:PSS substrates at 1750 rpm for 60 s to obtain the donor layer of about 70 nm. The BTP-eC9 solution was spin-coated on the rotating PM6 donor layer at 2250 rpm for 60 s to obtain the acceptor layers of about 55 nm. Then, the active layers were treated with thermal annealing at 100 °C for 10 min. As for BHJ cells fabricated via spin coating using CF, the active layer was spin coated from 16.5 mg mL^−1^ CF solution (D:A = 1:1.2, 0.5% v/v 1-chloronaphthalene, CN) at 3750 rpm for 30 s. For G-BHJ devices, the donor was firstly deposited from 7.25 mg mL^−1^ CF solution at 3000 rpm for 30 s, followed by deposing the acceptor solution in 7.5 mg mL^−1^ with 0.5% v/v CN in acceptor solution at 2750 rpm for 30 s. Then, a thermal annealing treatment at 100 °C for 10 min was performed for both active layers. For thicker film fabrication for the BHJ and G-BHJ OSCs, the variation of thickness was controlled by changing the total concentration and the spin coating speed. To be detailed, for BHJ thick devices, the 300 nm thickness was given by spin coating the solution of 15 mg mL^−1^ at the spin speed of 2750 rpm, 400 nm, and 500 nm thicknesses were given by spin coating the solution of 20 mg mL^−1^ at the spin speed of 4000 rpm and 3000 rpm, respectively. For G-BHJ thick devices, the concentrations of donor and acceptor (D/A) are 18/18 mg mL^−1^, 300 nm-based active layer was realized at the speed of 1750/1750 rpm for D/A, 20/20 mg mL^−1^ of D/A can get 400 nm-thick and 500 nm-thick active layers at 3250 rpm/1750 rpm and 2750/2750 rpm, respectively. Then a thermal annealing at 100 °C for 10 min was performed. The thicknesses of the films were measured by a Bruker Dektak XT stylus profilometer. For the blade-coated devices, PM6 and BTP-eC9 were dissolved at the polymer concentration of 10 mg mL^−1^ and the acceptor concentration of 10 mg mL^−1^. Before blade-coating the active layer, 0.1%, 0.3%, and 0.5% 1,8-diiodooctane (v/v) was added into acceptor layer solutions. An automatic wire-bar coater (RK PrintCoat Instruments, K paint type) was empolyed in a humidity-control ambient environment (30 ± 5% RH). Small droplet of active layer solution was dripped on the substrate and swiped linearly by an adjustable film applicator (BEVS 1806B/100) The blade speed was set as 20, 25, 30, 40, 50, and 60 mm s^−1^. The gap between the film applicator and substrate was set as 100 µm. When coating the active layer, the ITO-based substrate was maintained as 60 °C. A laminar nitrogen knife was installed right next to the substrate with the flow at an angle of 20° to the substrate. The as-prepared wet active layer film was gas-quenched by the nitrogen knife with the fixed nitrogen blow rate of 40 m s^−1^ (calibrated using the Testo 416 flowmeter), in order to facilitate solvent drying and film crystallization. Subsequently, the films were treated with the thermal annealing at 100 °C for 10 min.

For the optimal BHJ devices via blade coating, 16.5 mg mL^−1^ CF solution (*D*:*A* = 1:1.2, 0.5% v/v CN) was blade coated at the blade speed of 25 mm s^−1^. For the optimal G-BHJ devices via blade coating, 7.25 mg mL^−1^ CF solution of donor was blade coated at the blade speed of 25 mm s^−1^, 7.25 mg mL^−1^ CF solution of acceptor (0.5% v/v CN) was blade coated at the blade speed of 60 mm s^−1^. When coating the active layer, the ITO-based substrate was maintained at room temperature. The as-prepared wet active layer film was gas-quenched by the nitrogen knife with the fixed nitrogen blow rate of 40 m s^−1^. Then, a thermal annealing of 100 °C for 10 min was performed for all the films.

Deposition of interface layer in the blade-coated devices are the same as that in the spin-coated devices. As for G-BHJ OSCs based on PM6:IT-4F and PCE-10:IEICO-4F systems, optimal active layers were spin-coated from XY solutions with a *D*:*A* weight ratio of 1:1 for PM6:IT-4F and 1:1.5 for PCE-10:IEICO-4F with a small amount of additive in acceptor solutions (0.5% DIO for IT-4F, 2% CN for IEICO-4F), followed by a post thermal annealing of 100 °C for 10 min. A thin layer (~5 nm) of PFN-Br was spin coated on the active layer at 3000 rpm for 30 s. Finally, the Ag (90 nm) electrode was deposited by thermal evaporation to complete the whole device with the active area of 0.04 cm^2^, which is typical defined by a metal mask with an aperture aligned with the device area.

The current density-voltage (*J–V*) characteristics were measured in the glovebox with a Keithley 2400 measure unit under 1 sun, AM 1.5 G spectra (100 mW cm^−2^) from a solar simulator (Enli Tech. Co., Ltd., Taiwan). The light intensity was calibrated with a 20 mm × 20 mm monocrystalline silicon reference cell with KG5 filter (Enli Tech. Co., Ltd., Taiwan). The *J*–*V* curves are measured along the forward scan direction from −1.5 to 1.5 V, with a scan step of 20 mV with delay time of 1 ms. The EQE was measured by solar cell spectral response measurement system QE-R3-011 (Enli Tech. Co., Ltd., Taiwan). The light intensity at each wavelength was calibrated with a standard single-crystal Si photovoltaic cell.

### DP-XPS measurements

XPS experiments performed on the Thermo Scientific Nexsa using 12 kV cathode biased Al Ka radiative source. The base pressure in the analysis chamber was about 5 × 10^−10^ mbar. Depth profiling tests were conducted by using an Ar^+^ sputtering gun operated at 1 keV with raster size of 1 mm × 1 mm.

### TOF-SIMS measurements

TOF-SIMS was performed using a TOF-SIMS instrument (ION TOF TOF-SIMS V), where a 10 keV Ar_1500_^+^ cluster ion source was used for sputtering and a 25 keV Bi_3_^+^ pulsed primary ion beam was used for the analysis. The area of analysis was 150 × 150 µm^2^.

### SCLC measurements

The hole and electron mobility of the D/A blend film was measured using the space charge limited current (SCLC) method. The structure of hole-only device and electron-only mobility device is ITO/PEDOT: PSS/Active layer/Au and ITO/ZnO/ Active layer/Ag, respectively. The mobility was determined by fitting the dark current to the model of a single carrier SCLC, which is described by the equation:

$$J=\frac{9}{8}{\varepsilon }_{o}{\varepsilon }_{{{{\rm{r}}}}}{\mu }_{{{{\rm{h}}}}}\frac{{V}^{2}}{{d}^{3}}$$

where *J* is the current, *μ*_h_ is the charge mobility at zero field, *ε*_o_ is the free-space permittivity, *ε*_r_ is the relative permittivity of the material, *d* is the thickness of the active layer, and *V* is the effective voltage *V*–*V*_bi_, where is the built-in voltage. The hole-mobility and electron-mobility can be fit from the Ln(*J*L^3^/*V*^2^) – (*V*/L)^0.5^ curves and Ln(*J*) – Ln(*V*) curves respectively.

### GI-XRD characterization

GI-XRD measurements were carried out with a Xeuss 2.0 SAXS/WAXS laboratory beamline using a Cu X-ray source (8.05 keV, 1.54 Å) and Pilatus3R 300 K detector. The critical incidence angle is 0.2°. For the angle-dependent GI-XRD, 0.15°, 0.20°, and 0.25° were chosen.

### Photoluminescence characterizations

The steady PL spectra were measured by the FLS 920 (Edinburgh Instruments, Ltd) with excitation at 635 nm.

### Reporting summary

Further information on research design is available in the Nature Research Reporting Summary linked to this article.

## Supplementary information

Supplementary Information

Solar Cells Reporting Summary

## Data Availability

The authors declare that the experimental data that support the findings of this paper are available within the article and its Supplementary Information files. The source data are provided as a Source Data file. Other findings in this study are available from the corresponding authors upon reasonable request. Source data are provided with this paper.

## Source data

Source Data
