# Supplementary material for: Graded bulk-heterojunction enables 17% binary organic solar cells via nonhalogenated open air coating
Source: Nat Commun. 2021 Aug 10;12:4815. doi: 10.1038/s41467-021-25148-8 (PMC8355148; doi:10.1038/s41467-021-25148-8)
Supplement: Supplementary file 1 — Supplementary Information [file 41467_2021_25148_MOESM1_ESM.pdf]

Supplementary Materials for

Graded Bulk-Heterojunction Enables 17% Binary Organic Solar Cells via Nonhalogenated Open Air Coating

Ying Zhang[1], Kuan Liu[1*], Jiaming Huang[1], Xinxin Xia[2], Jiupeng Cao[3], Guangming Zhao[3], Patrick W.K. Fong[1], Ye Zhu[3], Feng Yan[3], Yang Yang[4], Xinhui Lu[2], Gang Li[1]*

[1]The Department of Electronic and Information Engineering, Research Institute for Smart Energy (RISE), The Hong Kong Polytechnic University, Hong Kong 999077, China.

[2]Department of Physics, The Chinese University of Hong Kong, New Territories, China.

[3]The Department of Applied Physics, The Hong Kong Polytechnic University, Hong Kong 999077, China.

[4]The Department of Materials Science and Engineering, UCLA, Los Angeles CA, USA.

*Correponding author. Email: kuan-lk.liu@polyu.edu.hk (K. L.);
gang.w.li@polyu.edu.hk (G. L.)

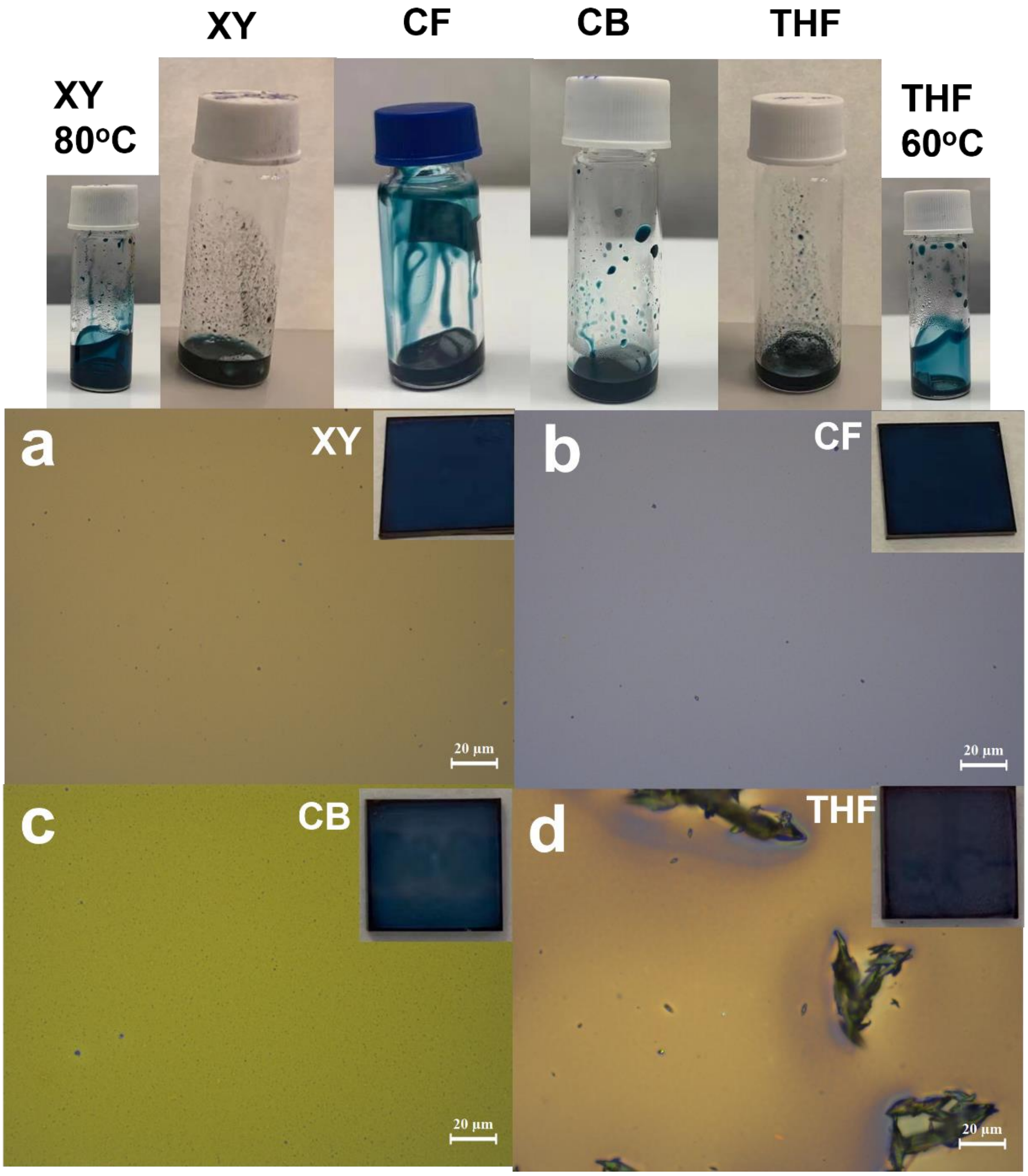

Supplementary Fig. 1 Solubility tests of the NFA in different processing solvents and optical images of SD films rom various solvents.

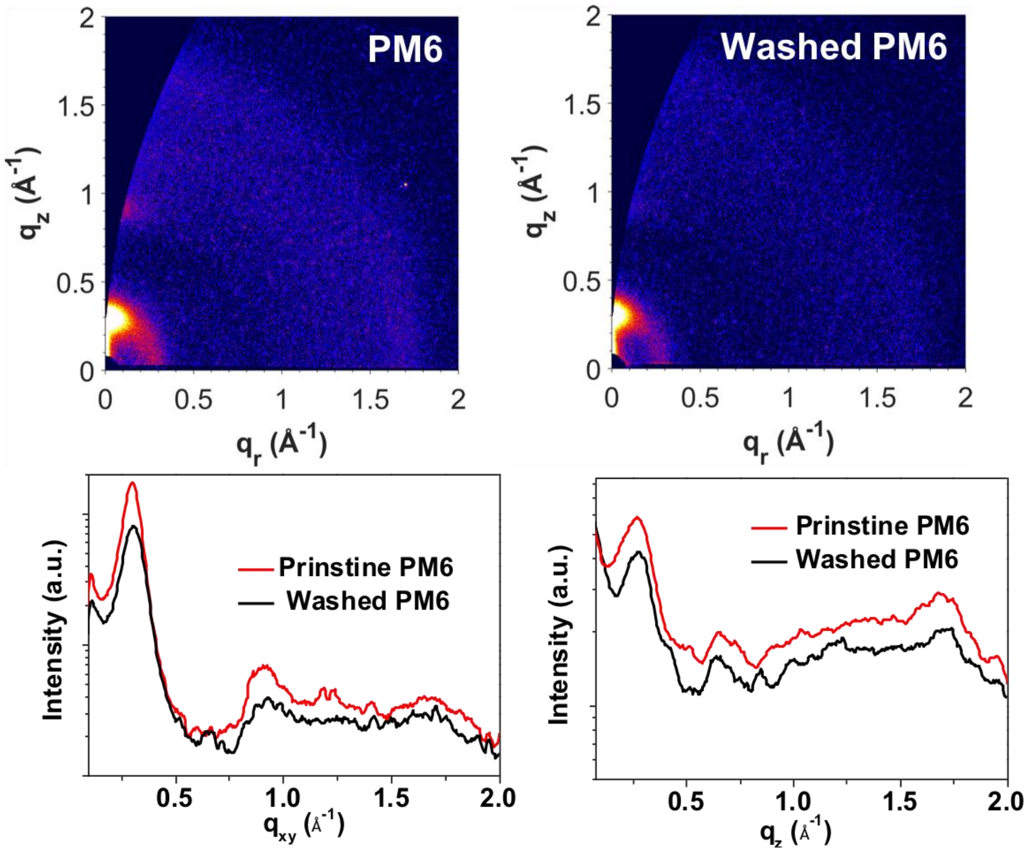

Supplementary Fig. 2 The corresponding 2D GIWAXS patterns of the neat PM6 film and the washed PM6 by XY and the corresponding line profiles in the in-plane and out of plane directions of PM6 film and the washed PM6 film by XY.

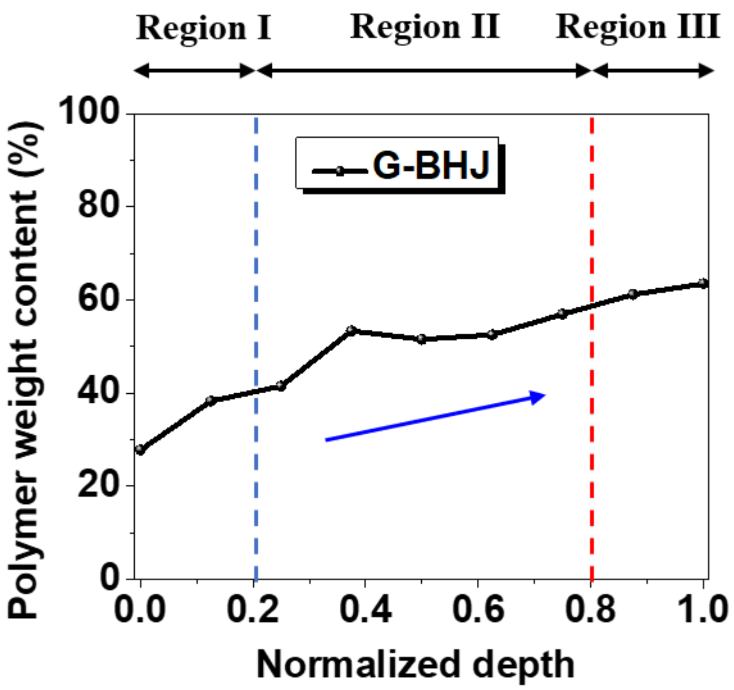

Supplementary Fig. 3 Variation of polymer weight content of optimal G-BHJ films from CF solution across the whole film.

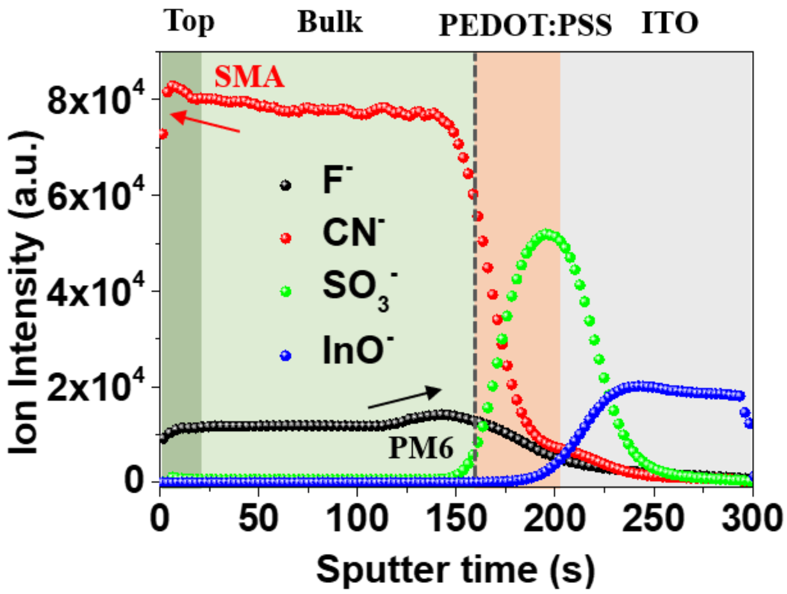

Supplementary Fig. 4 ToF-SIMS ion of G-BHJ films processed by XY with DIO additive as a function of sputter time, where fluoro (F^-), cyano group (CN^-) and sulfonic acid group (SO_3^-) and InO^- ions were characteristic mass fragments of PM6, the BTP-eC9 and PEDOT:PSS and ITO. Thus, these ions can be detected to map the donor and acceptor distribution throughout the whole film. All the samples were prepared on the ITO/PEDOT:PSS substrates.

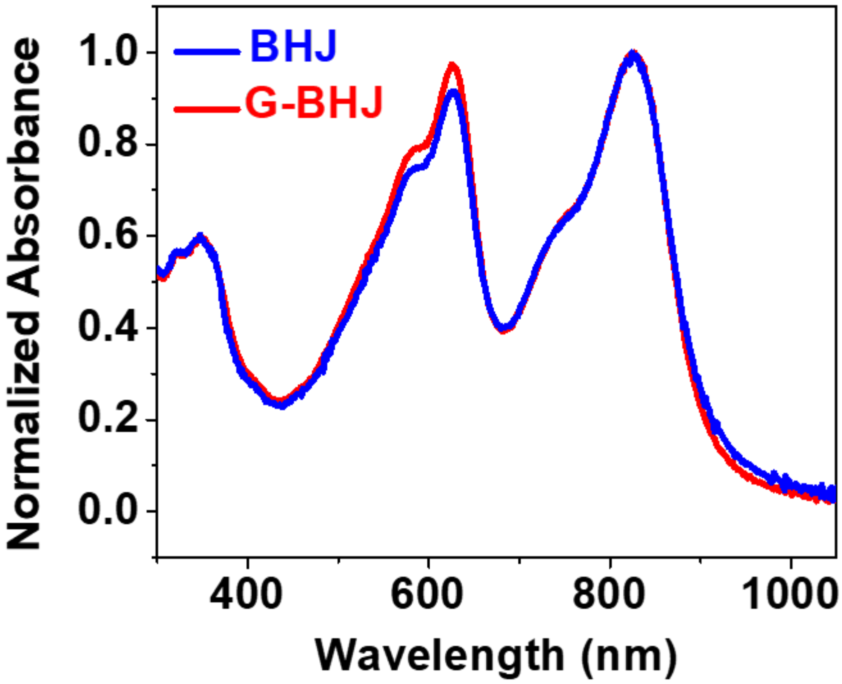

Supplementary Fig. 5 The normalized UV-vis absorption of BHJ and G-BHJ films. PM6 and BTP-eC9 dominate the film absorption profile in the G-BHJ and BHJ films, displaying the similar absorption spectra in the ranges of at 470-680 nm and 700-950 nm, respectively.

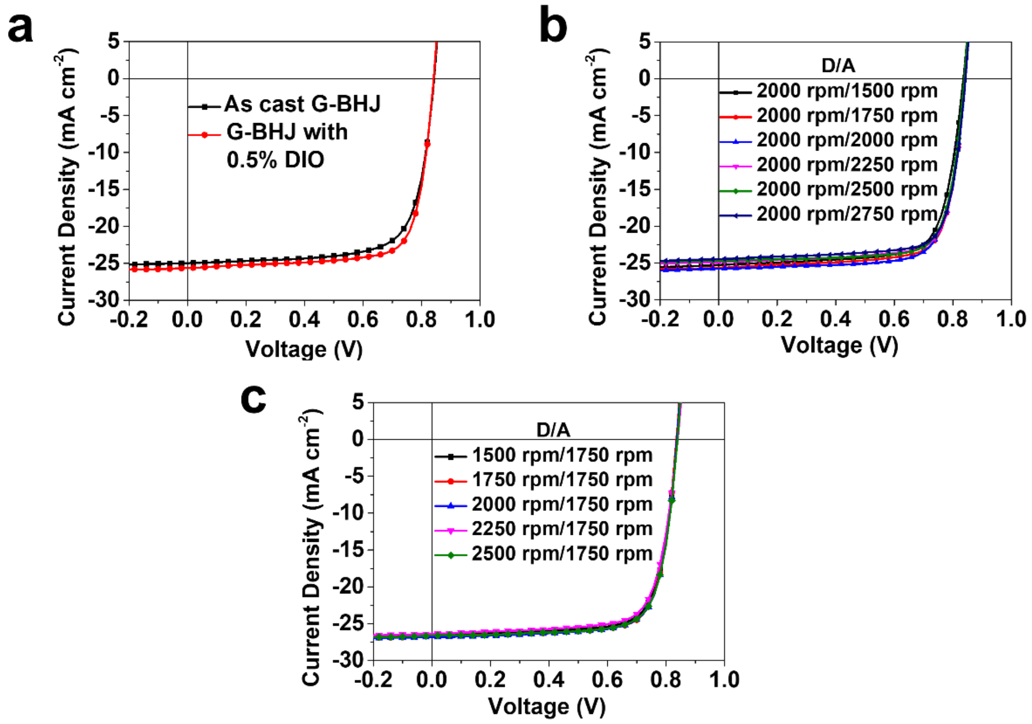

Supplementary Fig. 6 *J*-*V* curves of G-BHJ OSCs a) with different DIO amount, b) and c) under varied D/A spin coating speeds.

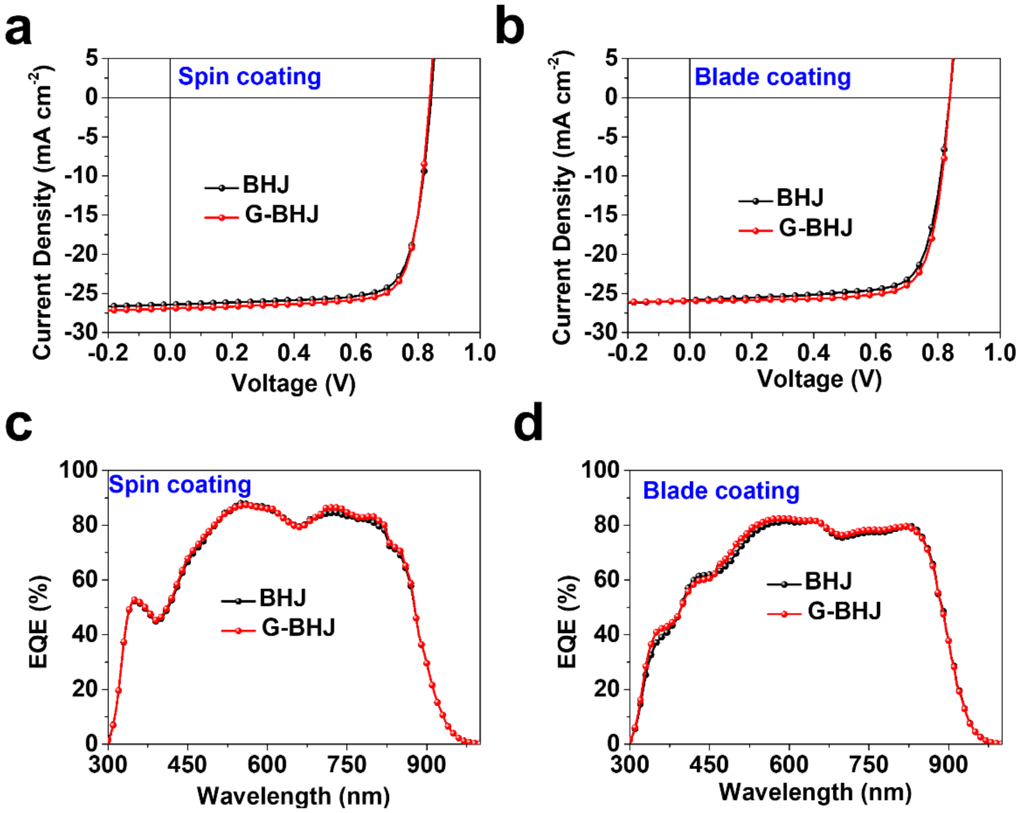

Supplementary Fig. 7 *J-V* curves of BHJ and G-BHJ OSCs via a) spin coating and b) via blade coating. c) EQE curves of BHJ and G-BHJ OSCs via c) spin coating and d) via blade coating.

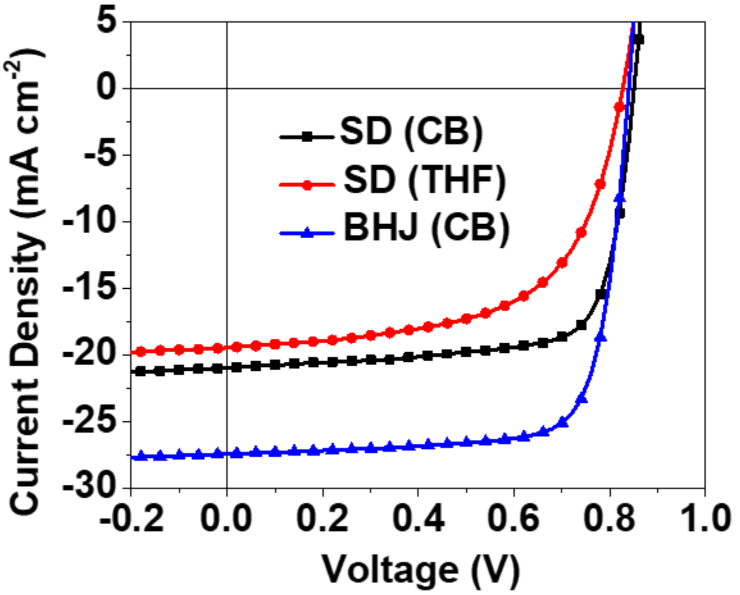

Supplementary Fig. 8 *J-V* curves of optimal SD OSCs using THF and CB solvnets for the upper layer as well as BHJ OSC using CB as the processing solvent.

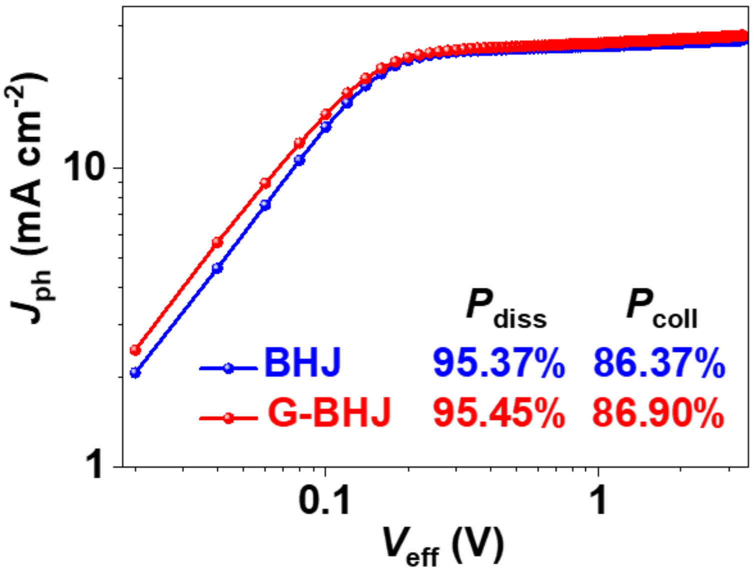

Supplementary Fig. 9 Photocurrent density (J_{ph}) plotted versus effective bias for optimized BHJ and G-BHJ devices.

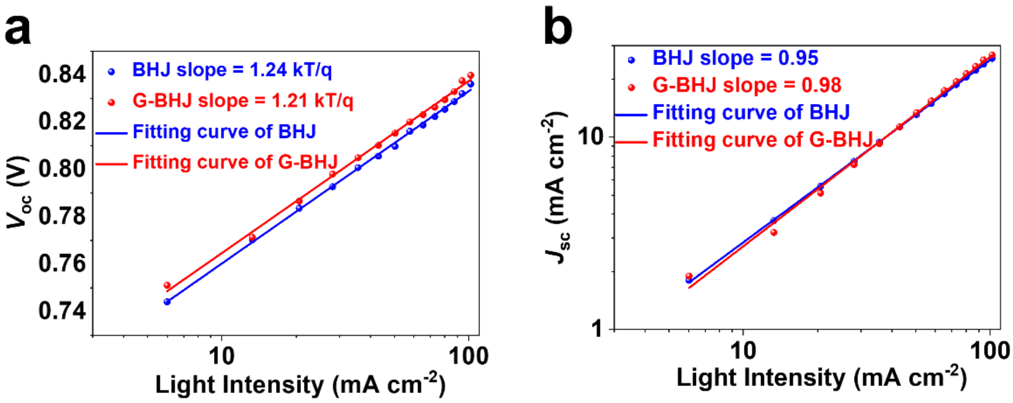

Supplementary Fig. 10 Photocurrent density (J_{ph}) plotted versus effective bias for optimized BHJ and G-BHJ devices. Light intensity dependent a) V_{OC} and b) J_{SC} for optimized BHJ and G-BHJ devices.

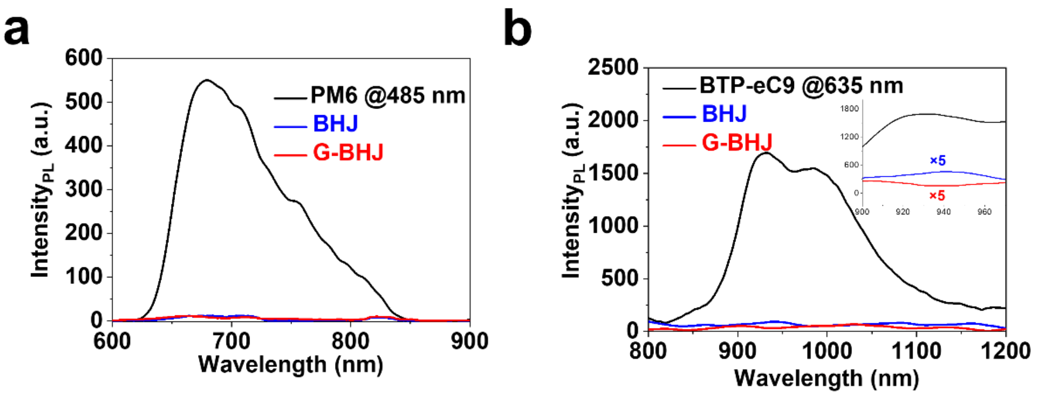

Supplementary Fig. 11 a) PL spectra the neat PM6, the optimal BHJ and G-BHJ blends, excited at 485 nm. b) PL spectra the neat BTP-eC9, the optimal BHJ and G-BHJ blends, excited at 635nm.

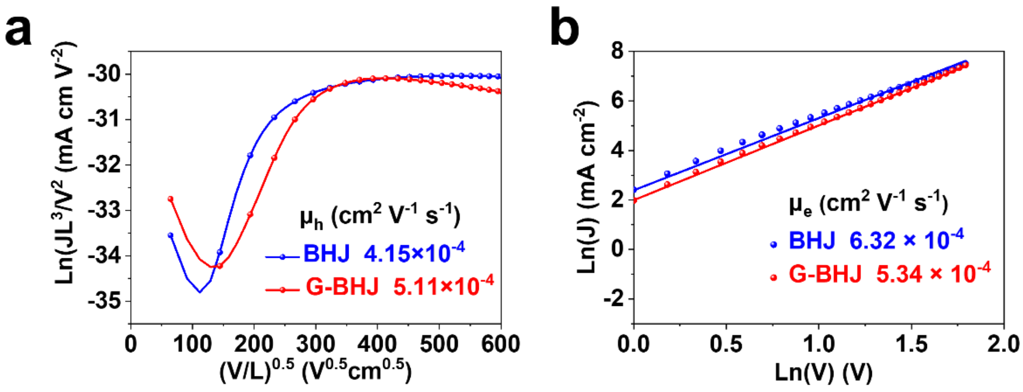

Supplementary Fig. 12 Plots obtained from the a) hole-only and b) electron-only devices based the optimal BHJ and G-BHJ devices via spin coating.

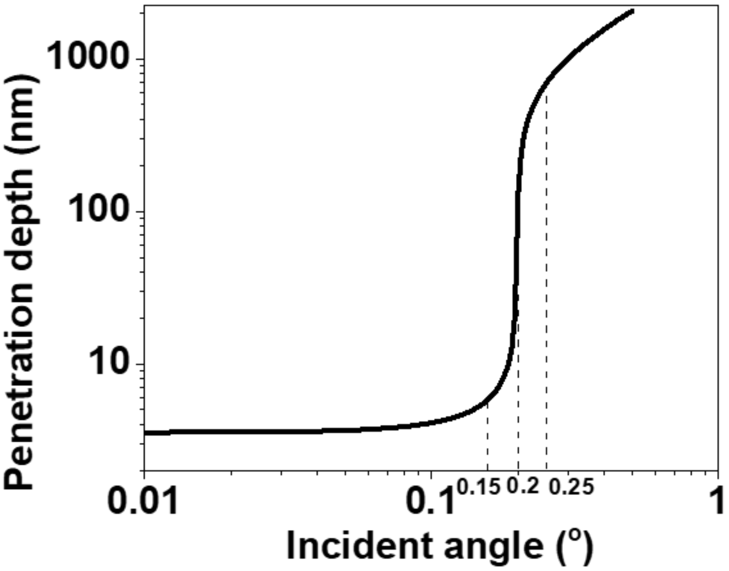

Supplementary Fig. 13 The theoretical penetration depth of X-ray through PM6: BTP-eC9 thin film as versus incident angle.

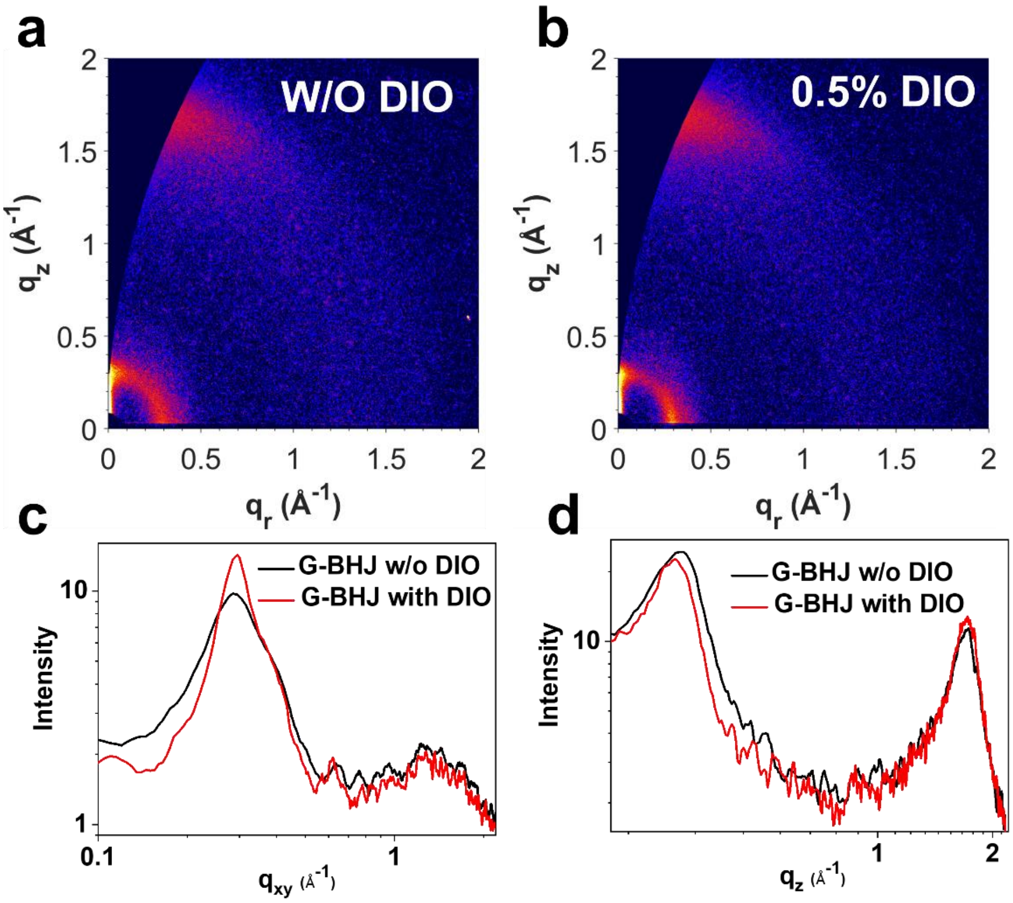

Supplementary Fig. 14 Line profiles in the a) IP and b) OOP directions of G-BHJ films via spin coating with and without DIO additive.

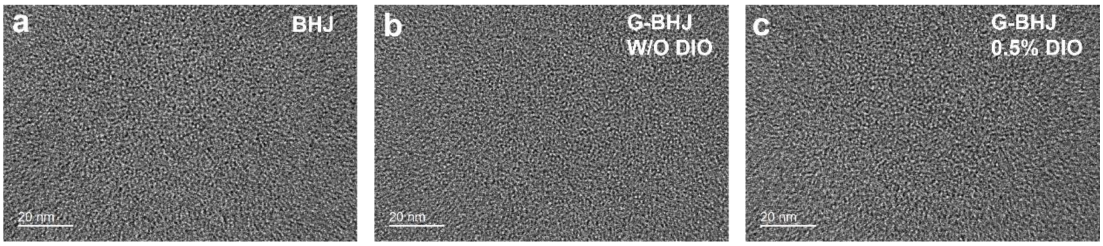

Supplementary Fig. 15 TEM images of a) theoptimal BHJ film, b) G-BHJ film without DIO and c) the optimal G-BHJ film via spin coating.

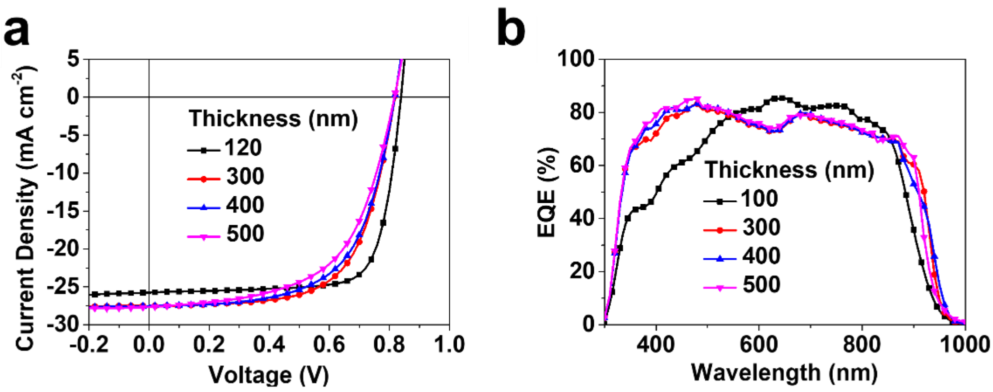

Supplementary Fig.16 a) *J*-*V* and b) EQE curves of the optimal BHJ OSCs via spin coating with different active layer thickness.

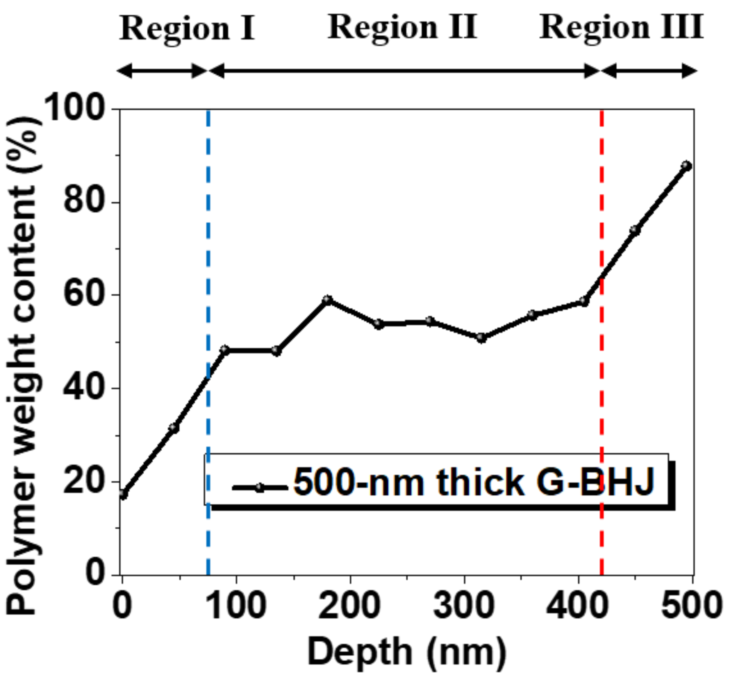

Supplementary Fig. 17 Polymer weight content of G-BHJ thick film of 500 nm at different depths.

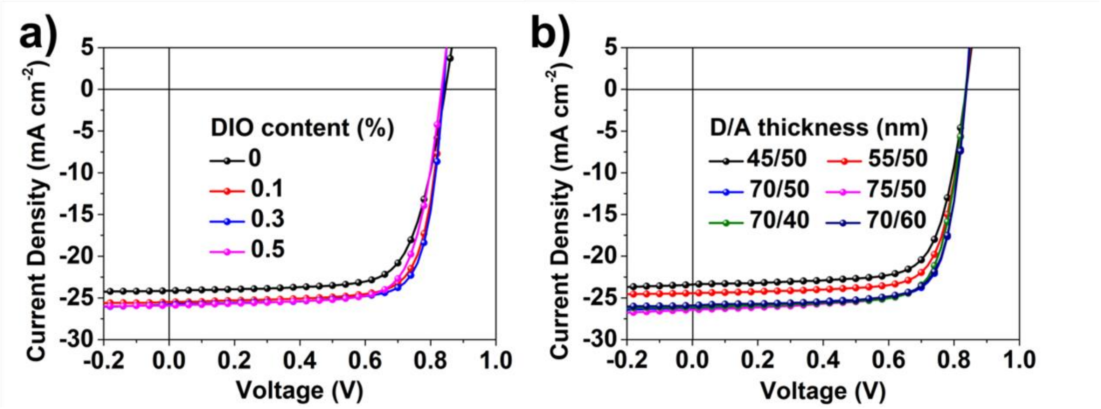

Supplementary Fig. 18 *J-V* curves of a) the blade-coated G-BHJ OSCs via spin coating with different amount of DIO additive and b) with varied D/A thickness.

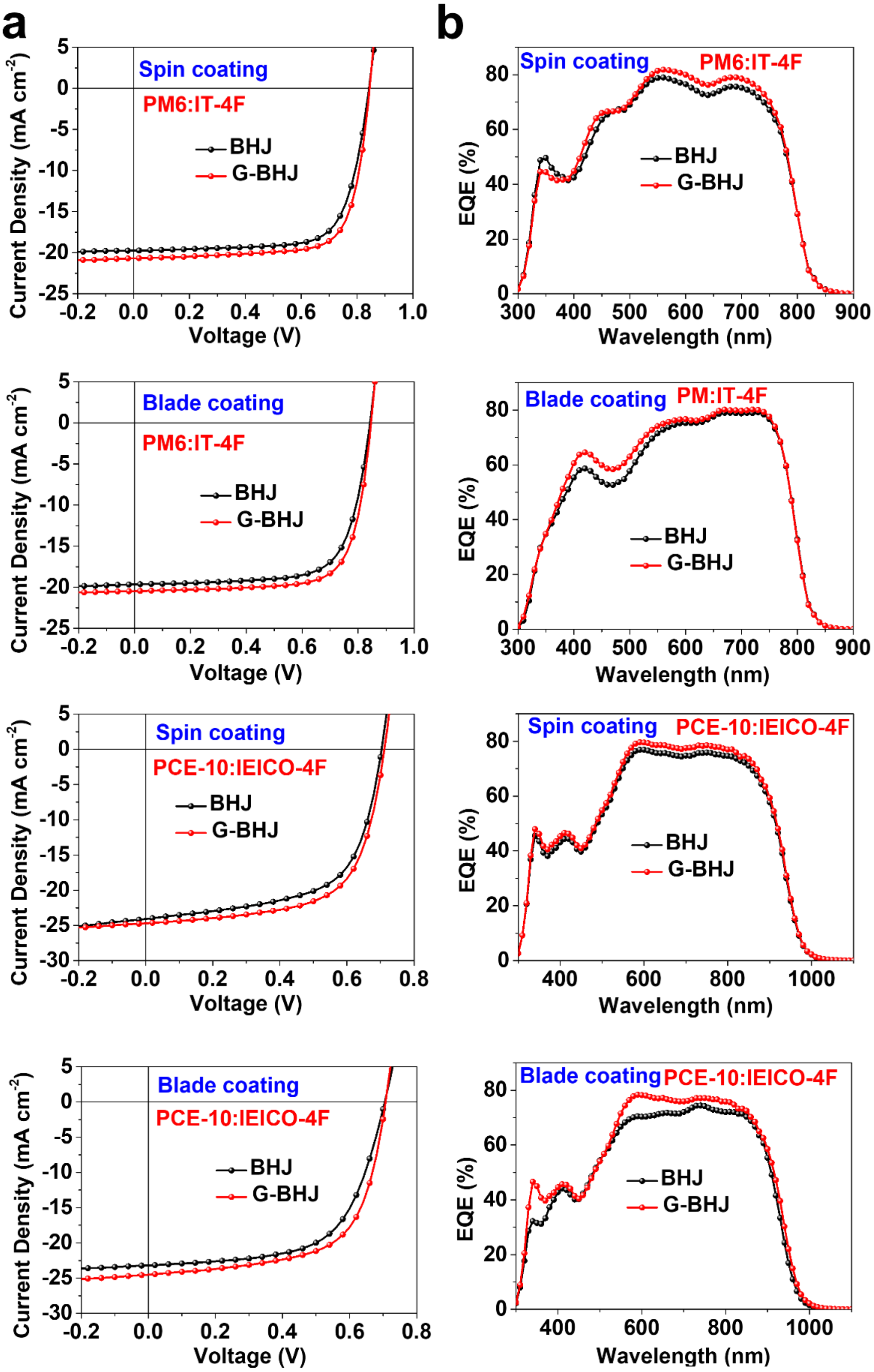

Supplementary Fig. 19 a) *J-V* characteristics and b) the corresponding EQE spectra for PM6:IT-4F and PCE-10:IEICO-4F based BHJ and G-BHJ OSCs processed by spin coating and blade coating methods.

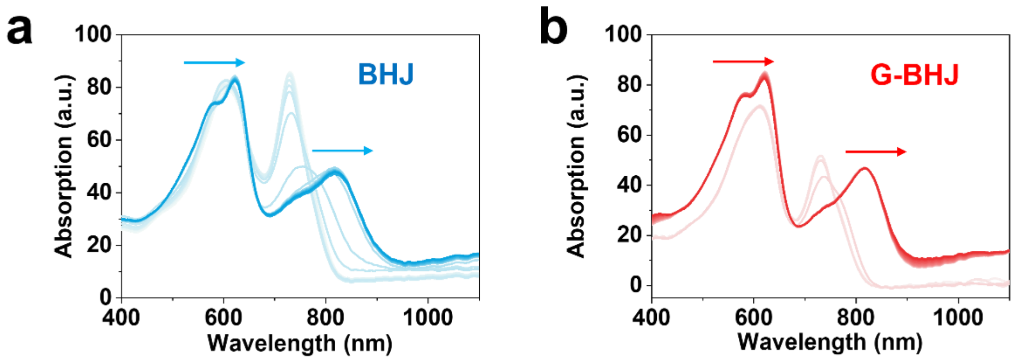

Supplementary Fig. 20 The *in situ* UV-vis absorption line profiles of the a) optimal BHJ and b) G-BHJ films via blade coating.

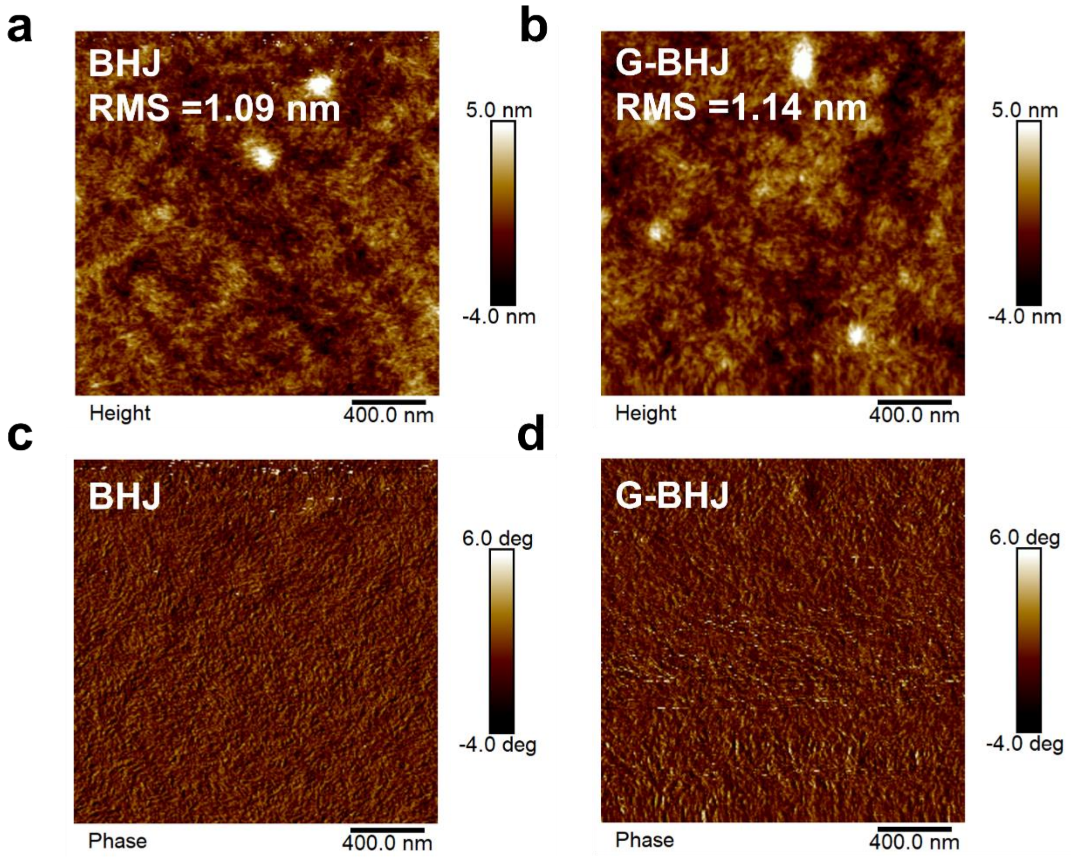

Supplementary Fig. 21 The height images of a) optimal BHJ and b) G-BHJ films via blade coating. The phase images of c) optimal BHJ and d) G-BHJ films via blade coating.

Supplementary Table 1. Polymer weight content of optimal BHJ, G-BHJ w/o DIO and optimal G-BHJ films with DIO via spin coating in different depth.

Deposition methods	Normalized Depth	Atomic ratio of O/F	Polymer weight content (wt%)
Optimal BHJ	0	2.50	34.0
	0.13	1.84	47.8
	0.25	1.78	49.7
	0.37	1.76	50.3
	0.62	1.80	49.0
	0.75	1.63	55.0
	0.67	1.89	46.4
	0.86	1.84	47.8
	1.00	2.04	42.5
G-BHJ w/o DIO	0	5.10	15.8
	0.11	2.98	28.0
	0.22	2.61	32.3
	0.33	2.01	43.2
	0.44	2.02	42.9
	0.56	1.83	48.1
	0.67	1.76	50.3
	0.78	1.45	63.1
	0.89	1.44	63.6
	1.00	1.25	75.5
Optimal G-BHJ	0	3.26	25.4
	0.11	2.57	32.9

0.22	2.19	39.3
0.33	1.74	51.0
0.44	1.65	54.2
0.56	1.55	58.3
0.67	1.58	57.1
0.78	1.55	58.3
0.89	1.51	60.1
1.00	1.39	66.4

Supplementary Table 2. Polymer weight content of optimal G-BHJ processed by CF with addition of DIO via spin coating in different depth.

Deposition methods	Normalized Depth	Atomic ratio of O/F	Polymer weight content (wt%)
Optimal BHJ	0	3.00	27.8
	0.12	2.24	38.3
	0.25	2.09	41.4
	0.37	1.67	53.4
	0.50	1.72	51.7
	0.62	1.69	52.7
	0.75	1.58	57.0
	0.88	1.49	61.1
	1.00	1.44	63.6

Supplementary Table 3. Photovoltaic parameters for G-BHJ OSCs with or without DIO via spin coating.

Processing additive DIO (%)	V_{OC} [V]	J_{SC} [mA cm^{-2}]	FF (%)	PCE_{max} [%]
0	0.843	24.96	0.73	15.36
0.5	0.841	25.65	0.76	16.40

Supplementary Table 4. Devices parameters of G-BHJ OSCs via spin coating based on PM6 and BTP-eC9 with different acceptor thickness. 0.5% DIO was added into BTP-eC9 solution.

Speed (rpm)/Thickness (nm)		V_{OC} [V]	J_{SC} [mA cm^{-2}]	FF (%)	PCE_{max} [%]
Donor	Acceptor				
2000/72	1500/65	0.837	25.08	0.75	15.75
2000/72	1750/55	0.841	25.74	0.76	16.45
2000/72	2000/53	0.841	25.65	0.76	16.40
2000/72	2250/50	0.842	24.83	0.77	16.10
2000/72	2500/48	0.840	24.73	0.77	16.00
2000/72	2750/43	0.843	24.43	0.77	15.86

Supplementary Table 5. Devices parameters of G-BHJ OSCs via spin coating based on PM6 and BTP-eC9 with different donor thickness. 0.5% DIO was added into BTP-eC9 solution.

Speed (rpm)/Thickness (nm)		V_{OC} [V]	J_{SC} [mA cm^{-2}]	FF (%)	PCE_{max} [%]
Donor	Acceptor				
1500/83	1750/55	0.837	26.47	0.76	16.82
1750/70	1750/55	0.836	26.61	0.77	17.13
2000/72	1750/55	0.836	26.81	0.76	17.11
2250/65	1750/55	0.840	26.30	0.75	16.57
2500/62	1750/55	0.840	26.70	0.76	17.05

Supplementary Table 6. Device performances of BHJ and G-BHJ OSCs via spin-coating and blade-coating processes using CF as the processing solvent under the illumination of an AM 1.5G solar simulator, 100 mW cm^{-2}.

	V_{OC} [V]	J_{SC} [mA cm^{-2}]	FF	PCE_{max} (PCE_{avg})[a] [%]	$J_{calc.}$[b]
BHJ[c]	0.842	26.44	0.767	17.08 (16.95±0.08)	25.89
G-BHJ[c]	0.839	26.91	0.777	17.54 (17.27±0.16)	26.09
BHJ[d]	0.838	25.90	0.751	16.30 (16.24±0.08)	24.98
G-BHJ[d]	0.838	26.00	0.770	16.78 (16.69±0.09)	25.22

The errors are defined as standard deviation. [a]Average PCE from 10 independent cells. [b]denotes integrated J_{sc} from the EQE curves. [c]The optimal OSCs via spin coating. [d]The optimal OSCs via blade coating.

Supplementary Table 7. Photovoltaic parameters for optimal SD and BHJ OSCs using different processing solvents.

Processing methods/ solvent	V_{OC} [V]	J_{SC} [mA cm^{-2}]	FF (%)	PCE_{max} [%]
SD/THF	0.826	19.50	0.601	9.68
SD/CB	0.850	20.97	0.74	13.19
BHJ/CB	0.839	27.46	0.763	17.58

Note: donor and acceptor hardly dissolve in the THF solvent, so no device data shown here.

Supplementary Table 8. J_{ph}, J_{sat}, G_{max} *and* J_{ph}/J_{sat} values of typical OSCs via spin coating.

Deposition method	J_{ph} (mA cm^{-2}) [a]	J_{ph} (mA cm^{-2})[b]	J_{sat} (mA cm^{-2})	J_{ph}/J_{sat} [a] (%)	J_{ph}/J_{sat} [b] *(%)*
BHJ	25.75	23.32	27.00	95.37	86.37
G-BHJ	26.65	24.26	27.75	96.03	87.42

[a]short-circuit condition, [b]maximal power output condition. J_{sat} is the reverse saturation current, i.e., the saturated/unchanged diode current when "large" reverse bias voltage was applied.

Supplementary Table 9. Hole and electron mobility of all hole-only and electron devices of neat PM6, neat BTP-eC9, the optimal BHJ and G-BHJ based devices via spin coating.

Blend	μ_h ($cm^2\ V^{-1}\ s^{-1}$)	μ_e ($cm^2\ V^{-1}\ s^{-1}$)	μ_e/μ_h
Neat PM6	2.7×10^{-4}	-	-
Neat BTP-eC9	-	4.73×10^{-4}	-
BHJ	4.15×10^{-4}	6.32×10^{-4}	1.52
G-BHJ	5.11×10^{-4}	5.34×10^{-4}	1.05

Supplementary Table 10. CCL of the lamellar staking (100) peak along IP direction of BHJ and G-BHJ films via spin coating.

Incident angle	Blends	Component	*q* [Å^{-1}]	*d*-spacing [Å^{-1}]	FWHM [Å^{-1}]	CCL [Å^{-1}]
0.15 ^{o}	BHJ	PM6	0.29	21.66	0.071	80.71
		BTP-eC9	0.38	16.53	0.104	54.32
	G-BHJ	PM6	0.29	21.66	0.074	80.71
		BTP-eC9	0.37	16.89	0.090	62.77
	G-BHJ (w/o DIO)	PM6	0.29	21.66	0.082	70.63
		BTP-eC9	0.36	17.44	0.221	25.68
0.20 ^{o}	BHJ	PM6	0.29	21.66	0.061	92.62
		BTP-eC9	0.38	16.53	0.179	31.56
	G-BHJ	PM6	0.29	21.66	0.050	113
		BTP-eC9	0.37	16.89	0.172	32.84
	G-BHJ (w/o DIO)	PM6	0.28	22.43	0.068	83.09
		BTP-eC9	0.36	17.44	0.204	27.67
0.25 ^{o}	BHJ	PM6	0.29	21.66	0.060	94.17
		BTP-eC9	0.36	17.44	0.170	33.23
	G-BHJ	PM6	0.29	21.66	0.055	102
		BTP-eC9	0.36	17.44	0.182	31.04
	G-BHJ(w/o DIO)	PM6	0.28	22.43	0.073	77.39
		BTP-eC9	0.39	16.10	0.204	27.67

Supplementary Table 11. CCLs of the π- π staking (010) peak of BHJ and G-BHJ films via spin coating.

Incident angle	Blends	*q* [Å⁻¹]	*d*-spacing [Å⁻¹]	FWHM [Å⁻¹]	CCL [Å⁻¹]
0.15 °	BHJ	1.74	3.61	0.290	19.48
	G-BHJ	1.70	3.69	0.274	20.62
	G-BHJ (w/o DIO)	1.72	3.65	0.282	20.04
0.20 °	BHJ	1.73	3.63	0.274	20.62
	G-BHJ	1.72	3.65	0.284	19.89
	G-BHJ (w/o DIO)	1.73	3.63	0.300	18.83
0.25 °	BHJ	1.73	3.63	0.287	19.67
	G-BHJ	1.72	3.65	0.260	21.73
	G-BHJ (w/o DIO)	1.73	3.63	0.300	18.83

Supplementary Table 12. Recent progress of efficient thick-film OSCs including both binary and ternary systems.

Material systems	Thickness (nm)	V_{OC} [V]	J_{SC} [mA cm^{-2}]	FF (%)	PCE_{max} [%]	Ref
PTQ10: IDTPC	400	0.913	17.90	61.3	10.0	[1]
PM7:MF2	500	0.953	19.20	54.9	10.04	[2]
PBDB-TF:IDTN	530	0.912	17.93	52	8.5	[3]
PM6:Y6:BTP-M	400	0.849	26.24	60.36	13.48	[4]
BTR:$PC_{71}BM$	300	0.93	13.95	69.6	9.6	[5]
PTQ10: IDIC	310	0.943	19.16	57.10	10.31	[6]
PTzBI-Si:N2200	520	0.84	16.4	63.2	8.7	[7]
PM6:SeTIC4Cl	300	0.75	22.8	66.1	11.3	[8]
PM6:F–2Cl	519	0.884	20.60	63	11.41	[9]
D18:Y6:$PC_{61}BM$	300	0.860	26.15	72.6	16.32	[10]
PBDB-T-2Cl:BT-4F:MF1	300	0.882	23.06	71.62	14.57	[11]
PBDB-TF: PBT(E)BTz:BTP-4Cl	300	0.854	26.61	61.61	14.00	[12]
PTB7-Th:BTR:$PC_{71}BM$	400	0.754	19.8	55.6	8.37	[13]
PBDB-T-2Cl: BTP-4F: $PC_{61}BM$	300	0.802	26.8	66.7	14.3	[14]
BTR:NITI: PC_{71} BM	300	0.94	19.50	73.83	13.63	[15]
PM6/BTP-eC9	300	0.830	26.89	72.8	16.25	This work
PM6/BTP-eC9	400	0.823	27.42	67.0	15.12	This work
PM6/BTP-eC9	500	0.821	27.31	64.1	14.37	This work

PM6:BTP-eC9	500	0.817	27.65	58.4	13.20	This work

Supplementary Table 13. Photovoltaic parameters of the optimal BHJ OSCs via spin coating with different active layer thickness.

Active layer thickness (nm)	V_{OC} [V]	J_{SC} [mA cm^{-2}]	FF	PCE_{max}	$J_{calc.}$ [mA cm^{-2}]
100±5	0.839	25.75	0.760	16.41	25.30
295±10	0.820	27.50	0.650	14.66	26.20
405±15	0.818	27.51	0.623	14.02	26.31
500±15	0.817	27.65	0.584	13.20	26.53

Supplementary Table 14. Polymer weight content of optimal 500 nm-thick G-BHJ via spin coating in different depth.

Depth (nm)	Atomic ratio of O/F	Polymer weight content (wt%)
0	4.68	17.3
45	2.68	31.4
90	1.83	48.1
135	1.83	48.0
180	1.54	58.9
225	1.66	53.8
270	1.64	54.6
315	1.75	50.6
360	1.61	55.8
405	1.54	58.7
450	1.27	74.0
495	1.11	87.5

Supplementary Table 15. The photovoltaic parameters of devices based on G-BHJ films with different D/A blade coating speeds under the illumination of an AM 1.5G solar simulator, 100 mW cm^{-2}.

Thickness of D/A (nm) (blade speed of D/A, mm/s)	V_{OC} [V]	J_{SC} [mA cm^{-2}]	FF	PCE$_{max}$
45/50 nm (20/60, mm/s)	0.837	23.51	0.73	14.36
55/50 nm (25/60, mm/s)	0.837	24.48	0.75	15.37
70/50 nm (30/60, mm/s)	0.837	26.16	0.76	16.64
75/50 nm (40/60 mm/s)	0.836	26.42	0.74	16.42
70/40 nm (50/40, mm/s)	0.836	26.09	0.75	16.42
70/60 nm (50/50, mm/s)	0.836	26.00	0.76	16.52

Supplementary Table 16. Photovoltaic properties of the G-BHJ OSCs via blade coating with different amount of DIO additive.

Amount of DIO [v/v, %]	V_{OC} [V]	J_{SC} [mA cm^{-2}]	FF	PCE_{max}
0	0.845	24.18	0.72	14.71
0.1	0.839	25.53	0.76	16.28
0.3	0.839	25.87	0.77	16.71
0.5	0.834	25.90	0.74	15.98

Supplementary Table 17. The performance parameters of the champion devices of BHJ and G-BHJ OSCs based on different active layers and processing techniques.

Active layer	Film type	Processing method	V_{OC} [V]	J_{SC} ($J_{calc.}$[a]) [mA cm^{-2}]	FF	PCE [%]
PM6+IT-4F	BHJ	Spin coating	0.843	19.74 (18.84)	0.730	12.15
		Blade coating	0.843	19.64 (18.76)	0.717	11.87
	G-BHJ	Spin coating	0.845	20.59 (19.60)	0.748	13.01
		Blade coating	0.846	20.40 (19.42)	0.743	12.81
PCE-10+IEICO-4F	BHJ	Spin coating	0.703	24.06 (23.61)	0.616	10.42
		Blade coating	0.705	23.21 (22.67)	0.617	10.10
	G-BHJ	Spin coating	0.711	24.70 (24.42)	0.642	11.28
		Blade coating	0.707	24.50 (24.02)	0.635	11.00

[a]The J_{calc} was calculated from the EQE spectrum.

Supplementary Note 1

Calculation of the compositions at vertical distribution

$$\text{PM6 weight content} = \frac{\text{Polymer weight}}{\text{Polymer weight} + \text{SMA weight}} \tag{1}$$

$$\text{PM6 weight content} = \frac{n_{\text{polymer}} M_{\text{polymer}}}{n_{\text{polymer}} M_{\text{polymer}} + n_{\text{SMA}} + M_{\text{SMA}}} \tag{2}$$

$$\text{PM6 weight content} = \frac{1}{1 + \frac{n_{\text{SMA}} + M_{\text{SMA}}}{n_{\text{polymer}} M_{\text{polymer}}}} \tag{3}$$

$$\frac{2n_{\text{polymer}} + 2n_{\text{SMA}}}{2n_{\text{polymer}}} = \frac{\text{O}}{\text{F}} \tag{4}$$

$$\frac{n_{\text{SMA}}}{n_{\text{polymer}}} = \frac{\text{O}}{\text{F}} - 1 \tag{5}$$

Therefore, according to euquations (1)-(5), we can deduce the polymer weight contens.

$$\text{PM6 weight content} = \frac{1}{1 + 1.3\left(\frac{\text{O}}{\text{F}} - 1\right)} \tag{6}$$

M_{SMA} is the molecular weight of BTP-eC9 (1572);

$M_{Polymer}$ is the molecular weight of repeat unit of PM6 (1218);

$n_{polymer}$ is the mole number of PM6; n_{SMA} is the mole number of repeat unit of BTP-eC9; O/F is the atom ratio of O/F. The O/F atom ratios were calculated by the integration of areas of corresponding XPS peaks and the atomic sensitivity factors. The simplified equation is O/F atom ratio = (O peak area/O sensitivity factor)/(F peak area/F sensitivity factor). Empirically derived set of atomic sensitivity factors for XPS can be acquired at http://202.38.64.11/~mams/escalab/sfactors.html.

Supplementary References

1. Luo, Z. et al. Side-chain impact on molecular orientation of organic semiconductor acceptors: high performance nonfullerene polymer solar cells with thick active layer over 400 nm. *Adv. Energy Mater*. **8**, 1800856 (2018).
2. Gao, W. et al. Thick-film organic solar Cells achieving over 11% efficiency and nearly 70% fill factor at thickness over 400 nm. *Adv. Funct. Mater.* **30**, 1908336 (2020).
3. Li, S. et al. Design of a new small-molecule electron acceptor enables efficient polymer solar cells with high fill factor. *Adv. Mater.* **29**, 1704051 (2017).
4. Zhan, L. et al. Layer-by-layer processed ternary organic photovoltaics with efficiency over 18. *Adv. Mater.* **33**, 2007231(2021).
5. Tang, H. et al. Delicate morphology control triggers 14.7% efficiency all-small-molecule organic solar cells. *Adv. Energy Mater.* **10,** 2001076 (2020).
6. Sun, C. et al. A low cost and high performance polymer donor material for polymer solar cells. *Nat.Commun.* **9**, 743 (2018).
7. Li, Z.-Y. et al. Achieving efficient thick film all-polymer solar cells using a green solvent additive. *Chinese J. Polym. Sci.* **38**, 323-331 (2019).
8. Wang, J. L. et al. Selenopheno[3,2-b]thiophene-based narrow-bandgap nonfullerene acceptor enabling 13.3% efficiency for organic solar cells with thickness-insensitive feature. *ACS Energy Lett.* **3**, 2967-2976 (2018).
9. Zhang, Y. et al. High performance thick-film nonfullerene organic solar cells with efficiency over 10% and active layer thickness of 600 nm. *Adv. Energy Mater.* **9,** 1902688 (2019).
10. Qin, J. et al. Over 16% efficiency from thick-film organic solar cells. *Sci. Bull.* **65,** 1979-1982 (2020).
11. Gao, J. et al. Over 14.5% efficiency and 71.6% fill factor of ternary organic solar cells with 300 nm thick active layers. *Energy Environ. Sci.* **13**, 958-967 (2020).
12. Zhang, Y. et al. A novel wide-bandgap polymer with deep ionization potential enables exceeding 16% efficiency in ternary nonfullerene polymer solar cells. *Adv. Funct. Mater.* **30,** 1910466 (2020).
13. Zhang, G. et al. High-performance ternary organic solar cell enabled by a thick active layer containing a liquid crystalline small molecule donor. *J. Am. Chem. Soc.* **139**, 2387-2395 (2017).
14. Ma, L. et al. A ternary organic solar cell with 300 nm thick active layer shows over 14% efficiency. *Sci. China Chem.* **63,** 21-27 (2019).
15. Zhou, Z. et al. High-efficiency small-molecule ternary solar cells with a hierarchical morphology enabled by synergizing fullerene and non-fullerene acceptors. *Nat. Energy* **3**, 952-959 (2018).
