# Supplementary material for: Graded bulk-heterojunction enables 17% binary organic solar cells via nonhalogenated open air coating
Source: Nat Commun. 2021 Aug 10;12:4815. doi: 10.1038/s41467-021-25148-8 (PMC8355148; doi:10.1038/s41467-021-25148-8)
Supplement: Supplementary file 2 — Solar Cells Reporting Summary [file 41467_2021_25148_MOESM2_ESM.pdf]

nature research

Corresponding author(s): Gang Li

Solar Cells Reporting Summary

Nature Research wishes to improve the reproducibility of the work that we publish. This form is intended for publication with all accepted papers reporting the characterization of photovoltaic devices and provides structure for consistency and transparency in reporting. Some list items might not apply to an individual manuscript, but all fields must be completed for clarity.

For further information on Nature Research policies, including our data availability policy, see Authors & Referees.

Experimental design

Please check: are the following details reported in the manuscript?

1. Dimensions

Item	Yes/No	Response
Area of the tested solar cells	☒ Yes ☐ No	The active area of the tested solar cells is 0.04cm^2, typical defined by a metal mask with an aperture aligned with the device area, as shown in Methods, section "Device fabrication and characterization".
Method used to determine the device area	☒ Yes ☐ No	You can find this information in the Methods, section "Device fabrication and characterization".

2. Current-voltage characterization

Item	Yes/No	Response
Current density-voltage (J-V) plots in both forward and backward direction	☐ Yes ☒ No	The organic solar cells here do not have hysteresis (forward and backward scan) problem, so we only focus on scanning the solar cells in the forward direction, and did not described explicitely.
Voltage scan conditions<br>*For instance: scan direction, speed, dwell times*	☒ Yes ☐ No	You can find this information in the Methods, section "Device fabrication and characterization".
Test environment<br>*For instance: characterization temperature, in air or in glove box*	☒ Yes ☐ No	Devices were characterized in In N2-filled glove box, described in Methods, section "Device fabrication and characterization".
Protocol for preconditioning of the device before its characterization	☐ Yes ☒ No	No preconditioning protocol.
Stability of the J-V characteristic<br>*Verified with time evolution of the maximum power point or with the photocurrent at maximum power point; see ref. 7 for details.*	☐ Yes ☒ No	Not tested, because the J-V stability issue is not a key claim in OPV as we understand, including our study.

3. Hysteresis or any other unusual behaviour

Item	Yes/No	Response
Description of the unusual behaviour observed during the characterization	☐ Yes ☒ No	Generally, there is no hysteresis in the organic solar cells. No unusual behaviour, such as Hysteresis, was observed in our device.
Related experimental data	☐ Yes ☒ No	No unusual behaviour was found for the related experimental data.

4. Efficiency

Item	Yes/No	Response
External quantum efficiency (EQE) or incident photons to current efficiency (IPCE)	☒ Yes ☐ No	See the Fig. 2, Fig. 5 and Fig. 6 in the revised manuscript and Supplementary Fig. 7, Supplementary Fig. 16 and Supplementary Fig. 19 in the revised supporting information.
A comparison between the integrated response under the standard reference spectrum and the response measure under the simulator	☒ Yes ☐ No	The integrated JSC values calculated from the EQE curves are consistent to those obtained from the J-V curves (within 2.5% error).
For tandem solar cells, the bias illumination and bias voltage used for each subcell	☐ Yes ☒ No	No tandem solar cells fabricated in our study.

5. Calibration

Light source and reference cell or sensor used for the characterization	[X] Yes [ ] No	The light source is a Class 3A solar simulator (Enli Tech. Co., Ltd., Taiwan). The light intensity was calibrated with a 20 mm×20 mm monocrystalline silicon reference cell with KG5 filter (purchased from Enli Tech. Co., Ltd., Taiwan, which has been Calibrated by authority party). The details can be found in Section "Methods".
Confirmation that the reference cell was calibrated and certified	[X] Yes [ ] No	The standard monocrystalline silicon reference cell with KG5 filter was purchased from Enli Tech. Co., Ltd., Taiwan and was calibrated and certified.
Calculation of spectral mismatch between the reference cell and the devices under test	[ ] Yes [X] No	We did not calculate the mismatch between the reference cell and the devices under test. The PI is the Co-first author of the ref. 1 (AFM 2006) below, in which he collaborated with NREL in 2005 and introduced the KG5 filtered mono-Si reference was to OPV field, due to closer spectral match. From our experience, the spectral mismatch should be limited to only a few %.

6. Mask/aperture

Size of the mask/aperture used during testing	[X] Yes [ ] No	A mask of 0.0289 cm^2 was defined by optical microscope.
Variation of the measured short-circuit current density with the mask/aperture area	[X] Yes [ ] No	The variation is within 0.2%.

7. Performance certification

Identity of the independent certification laboratory that confirmed the photovoltaic performance	[ ] Yes [X] No	As our work focuses on the novel film deposition, characterization methods and the corresponding mechanism, we have not certified the efficiency values.
A copy of any certificate(s) *Provide in Supplementary Information*	[ ] Yes [X] No	No available.

8. Statistics

Number of solar cells tested	[X] Yes [ ] No	The average PCE of OSC is obtained from 10 independent devices, as described in Table 1,Table 2 and Supplementary Table 6
Statistical analysis of the device performance	[X] Yes [ ] No	Statistical analysis of the device performances are provided in Table 1, Table 2 and Supplementary Table 6.

9. Long-term stability analysis

Type of analysis, bias conditions and environmental conditions *For instance: illumination type, temperature, atmosphere humidity, encapsulation method, preconditioning temperature*	[ ] Yes [X] No	Not tested yet, because the long time stability issue is not a key claim in our study.
